# Discretisation and continuity: The emergence of symbols in communication

**DOI:** 10.1016/j.cognition.2021.104787

**Published:** 2021-10

**Authors:** Robert Lieck, Martin Rohrmeier

**Affiliations:** Digital and Cognitive Musicology Lab, École Polytechnique Fédérale de Lausanne, 1015 Lausanne, Switzerland

**Keywords:** Communication games, Reinforcement learning, Language evolution, Symbol grounding, Discretisation

## Abstract

Vocal signalling systems, as used by humans and various non-human animals, exhibit discrete and continuous properties that can naturally be used to express discrete and continuous information, such as distinct words to denote objects in the world and prosodic features to convey the emotions of the speaker. However, continuous aspects are not always expressed with the continuous properties of an utterance but are frequently categorised into discrete symbols. While the existence of symbols in communication is self-evident, the emergence of discretisation from a continuous space is not well understood. In this paper, we investigate the emergence of discrete symbols in regions with a continuous semantics by simulating the learning process of two agents that acquire a shared signalling system. The task is formalised as a reinforcement learning problem with a continuous form and meaning space. We identify two causes for the emergence of discretisation that do not originate in discrete semantics: 1) premature convergence to sub-optimal signalling conventions and 2) topological mismatch between the continuous form space and the continuous semantic space. The insights presented in this paper shed light on the origins of discrete symbols, whose existence is assumed by a large body of research concerned with the emergence of syntactic structures and meaning in language.

## 1 Introduction

Vocal signalling systems exhibit both discrete and continuous properties across a wide range of species, from humans and monkeys to birds, dolphins, and whales (Eimas, Siqueland, Jusczyk, & Vigorito, 1971; Janik, 2009; Janik & Slater, 1997; Kuhl, 2004; Marino et al., 2007; Rendell & Whitehead, 2001; Wilbrecht & Nottebohm, 2003; Yurk, Barrett-Lennard, Ford, & Matkin, 2002). An utterance can be composed of one or more smaller acoustical units (e.g. a sentence composed of words), each having additional continuous properties (e.g. loudness, pitch, duration and timbre).

This combination of discrete and continuous components results in a unique tool for communication and coordination as both can be used to express an arbitrary amount of information while having complementary strengths: The discrete component allows for an infinite number of recombinations of the single acoustical units, which can be used to denote various things and circumstances in the world, such as objects or the presence of particular predators (Janik, Sayigh, & Wells, 2006; Ouattara, Lemasson, & Zuberbühler, 2009). However, discrete symbols can only approximately represent continuous information, such as shades of a colour or gradual changes in emotion. The continuous component, on the other hand, allows for a nuanced representation of gradual aspects, such as the emotional state of the speaker (Dimos, Dick, & Dellwo, 2015; Liebenthal, Silbersweig, & Stern, 2016; Scherer, 2003). In contrast, it does not provide absolute certainty about discrete aspects, such as whether a statement should be taken literally or ironically.

This interplay and coexistence of discrete and continuous aspects can not only be found in language but also in other communicative systems, such as music, where the continuous pitch space is discretised into scales with a number of distinct tones (Patel, 2003, Patel, 2010; Pearce & Rohrmeier, 2012; Sethares, 2005). Lying at the core of human cognition and communication, the integration of discrete and continuous aspects is as well important in research on artificial intelligence (Lake, Ullman, Tenenbaum, & Gershman, 2017; Lieck, 2018). From the perspective of language evolution, prior to the development of complex phonological and syntactic structures, the formation of the underlying discrete building blocks requires an explanation. This question is not only fundamental for the evolution of human language but also for communication in non-human animals. While the existence of discrete symbols in communication is self-evident, the reasons for their emergence have not been sufficiently investigated.

In this paper, we are concerned with the question *why discretisation occurs in regions with a continuous semantics*, instead of expressing all continuous aspects using continuous properties of the signal. That is, what causes a single contiguous semantic region to be split up and expressed using multiple discrete symbols, such as distinct colour words for the continuous space of colours (Berlin & Kay, 1969; Gibson et al., 2017; Kay, Berlin, Maffi, & Merrifield, 2003; Roberson, Davidoff, Davies, & Shapiro, 2004; Sandhofer & Smith, 1999; Steels & Belpaeme, 2005). This question concerns the early evolution of language, which by its very nature is difficult to study empirically. We therefore take a *synthetic* approach to understand the evolution of communication (Nolfi & Mirolli, 2010): By simulating the learning process of two agents that acquire a shared signalling system we investigate the development of continuous signalling conventions and possible reasons for the emergence of discretisation. We employ a setup that has been used in various other related works, with the important difference that we do not assume the existence of discrete symbols or categories at any point and all steps operate in continuous space. In order for our results to be applicable to the evolution of signalling systems in general, we confine ourselves to minimal and basic assumptions. The two-agent setup serves as a minimal test case and we expect our results to carry over to more complex scenarios involving multiple interacting agents. To the best of our knowledge, our work represents the first attempt to explain the emergence of discrete symbols in regions with continuous semantics based on simulations from first principles.

### 1.1 Terminology

We will now clarify the terminology used throughout the rest of the paper. The next section draws the connection to some of the terms commonly used in semiotics.

First, to be able to talk about communication on a general level, we will use the term *agent* to refer to any human, non-human animal, machine or any other entity that engages in an act of communication.

A *signal* or *form* is taken to be a physical quantity or object that is transmitted between the agents, carries information and thereby allows for communication between the agents. We generally use the term *form* because it is less technical and better connects to the terminology used in semiotics, as described in Section 1.2.

The *world* comprises anything that the agents perceive and that can be the subject of communication. Generally, the world comprises the agents themselves as well as the forms they exchange,^1^ however, to simplify the overall setup, we will assume a clean separation and exclude the agents and the forms from the world.

The *meaning* is the result of an agent interpreting a form it receives. In our experiments, we make the simplifying assumption that the meaning space is identical to the possible states of the world, that is, the meaning an agent attempts to communicate always corresponds to its perceived world in its entirety.

### 1.2 Semiotics

Concerning terminology as well as some general notions on communication, the field of semiotics is of particular relevance (Chandler, 2017; Nöth, 1990; Ogden & Richards, 1923). The central concept of a *sign* in semiotics has several characteristics that facilitate a connection to the approach developed in this paper.

First, while not providing exact mathematical definitions, signs in semiotics can have both a discrete or a continuous character.

Second, the concept of a sign is entangled with the idea of interpretation and signs are generally understood to comprise two (sometimes three) components. The left-hand side is associated to what an agent perceives (the *form*), while the right-hand side corresponds to how the form is interpreted (the *meaning*). Referring to the left-hand side as the *form* of a sign also accounts for situations where the same sign can appear in different forms. This conception is closely related to the ideas put forward in this paper and allows for a close connection to our formal definitions in Section 5.1.

Third, the bidirectional relation represented by a sign (in particular in Saussure's conception; Hurford, 1989) is reflected in our assumption of the sender and receiver policy being derived from the same underlying function (see Section 4.2.1).

The relation between the form and the meaning of a sign can have different (non-exclusive) characteristics, commonly classified as *iconic*, *indexical*, and *symbolic* (Chandler, 2017; Peirce, 1974, CP 2.275). An iconic relation is based on some kind of resemblance between the form and the meaning, while a symbolic relation is established by pure convention. An indexical relation is based on a physical or causal relation between the form and the meaning, however, this option is ruled out in our setup because of the clean separation of form and meaning space (i.e. forms are not part of the world).

In our experiments, we observe iconic relations whenever there is a continuous mapping between form and meaning space, such as in Fig. 2(a). This relation is *iconic* (in a somewhat technical sense) in that the form and the meaning space resemble each other (they have the same topology), so that a continuous variation of the form corresponds to an analogous variation in meaning (also cf. de Boer & Verhoef, 2012). We also observe symbolic relations when arbitrarily fragmented (discontinuous) mappings between form and meaning space are established, such as in Fig. 2(b). Such a fragmentation (discretisation) into symbolic relations breaks potential iconic relations. This provides a potential explanation why (especially in more technical fields) the term *symbolic* is frequently used synonymously with *discrete* (also cf. Feldman, 2012).

*Symbols*, as the term is used in this paper, generally have both a discrete and a continuous character: They are discrete in the sense that they are distinct from other symbols and continuous in that they may locally establish an iconic relation (also see Fig. 4). Purely discrete symbols arise as the atomic limit when a symbol has only a single form, so that the iconic mapping collapses into a single point.

### 1.3 Motivating example

Humans and many non-human animals communicate by means of auditory, visual, olfactory or haptic/tactile signals. Many of these signalling systems have discrete as well as continuous properties. Taking human language as an example, words can be combined to form sentences and each sentence can be pronounced in different ways. The discrete properties (how the words are combined) and the continuous properties (how the words are pronounced) together convey meaning with an astonishing level of detail. Consider the slight differences in meaning that a different pronunciation of the following sentences may convey:

**Figure:**
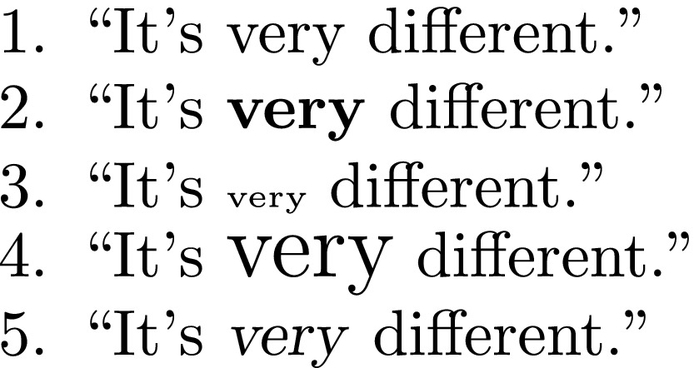

Visually, these examples are rendered by varying the values of three continuous font properties: weight, size, and slope. While many typesetting systems only offer a discrete subset of values for these properties, the nuances in pronunciation are abundant. In fact, in text media that are close to spoken language but do not allow for varying font properties, such as short messages, the urge for emulating the continuous properties of spoken language leads to an abuse of the discrete properties, such as character repetition for stretching a sound or capitalisation for loudness:

1. “It's veeeery different.”
2. “It's VERY different.”

The discrete and continuous properties of language are essential for a rich and nuanced communication and in linguistics they are intensively studied in the sub-fields of syntax and phonology, respectively (Robins, 2014).

In this paper, we are concerned with the relation between the discrete and the continuous properties of signalling systems. In particular, we are interested in two questions:

1. How did the discrete properties emerge given that the underlying space of forms is inherently continuous?
2. How can the discrete and the continuous properties be described in a unified way?

Before discussing related work on this topic and going into the technical details of the paper, we will give an overview of our main results.

## 2 Overview and summary

### 2.1 The emergence of discretisation

#### 2.1.1 Modal worlds

One possible explanation for the emergence of discretisation is that the world is inherently discrete. This idea does not necessarily conflict with the fact that our perception of the world is continuous. Even a continuous world may suggests an underlying discrete structure by being *modal* (Feldman, 2012): If our perception of the world can be described as a mixture of several well-discernable components, it can be effectively communicated by means of a discrete signalling system. The discrete properties of this system then reflect the modal structure of the world.

**Fig. 1:**
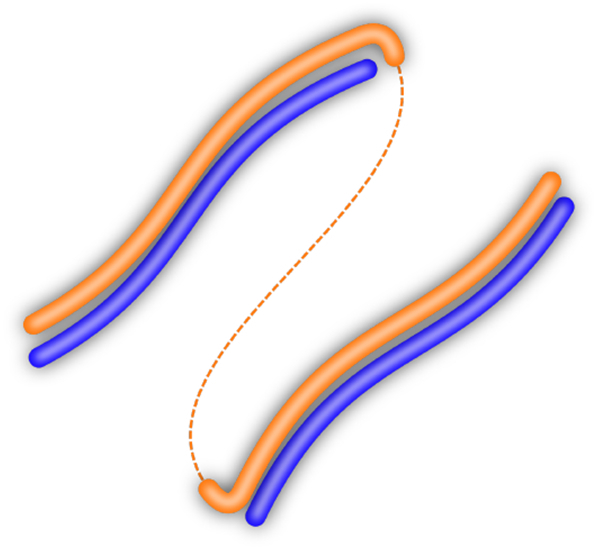
Worlds that exhibit distinct modes (blue lines) induce a discretisation in form space (orange), which has to be cut (dashed line) in order to be mapped to the world. (For interpretation of the references to colour in this figure legend, the reader is referred to the web version of this article.)

In this paper, we extend the scenario described by Feldman (2012) by assuming a continuous form space and showing how discretisation emerges in the case of modal worlds. Moreover, the resulting signalling system exhibits both discrete and continuous properties, which allows to not only refer to the separate modes but also represent more fine-grained differences *within* each of the modes. In our experiments, we use a setup where the world consists of two separate lines while the form space corresponds to a single contiguous line, as illustrated in Fig. 1.

However, in human language, we also observe discretisation in parts of the world that are inherently continuous, such as the colour spectrum. It is clear that the compositional nature of human language allows us to approximate continuous sub-spaces to an arbitrary precision using only discrete properties. We might for instance say: “Bricks are primarily red, with a hint of orange and a tiny little bit of grey.”^2^ Given the omnipresence of discrete entities in everyday communication (words, characters, symbols etc.) and our routine of using them to describe continuous aspects of the world, this seems to be an obvious approach. From an evolutionary perspective, however, it seems much more natural and effective to use continuous properties of the form to communicate continuous aspects of the world. Such as when communicating just *how* different something is by varying the way we pronounce the word “very” when saying: “It's very different.” So why are continuous aspects not always communicated using continuous properties?

It is well-conceivable that the discretisation of inherently continuous aspects of the world emerged as a secondary phenomenon: At an early stage of language evolution, relevant parts of the world may have suggested a discrete structure (in the sense of being modal), which resulted in a rudimentary signalling system with discrete in addition to continuous properties. These systems might have been similar to what we observe today in many non-human animals. In the subsequent development, this system might then also have been applied to communicate inherently continuous aspects of the world and served the purpose well enough to persist and further develop into the complex system of human language we observe today. This is a perfectly valid hypothesis, which we are neither trying to prove nor to refute in this paper. Instead we are asking a complementary question: Is a modal world the *only* way how discretisation could have emerged or are there other potential driving forces?

We will argue that there are at least two other potential reasons for discretisation in an entirely continuous and non-modal setting: sub-optimal conventions and topological mismatch.

#### 2.1.2 Sub-optimal conventions

**Fig. 2:**
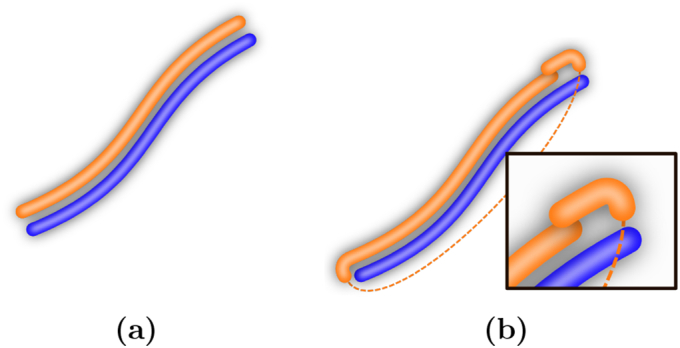
1D-1D-setting with a one-dimensional world (blue) and a one-dimensional form space (orange). (a): Optimal mapping. (b): Sub-optimal mapping, where one end of the meaning space is mapped to the “wrong” end of the form space, resulting in a fragmented form space with a cut (dashed line). (For interpretation of the references to colour in this figure legend, the reader is referred to the web version of this article.)

The first reason is a pragmatic one: Even if there exists a continuous mapping between forms and meanings that would be the optimal solution for communication, this solution might not always be found. After all, human language was not designed at the drafting table and then magically injected into our heads. Instead, the way we communicate today is the result of an evolutionary and social process, which is by no means guaranteed to have converged to the optimal solution. We investigate this scenario by simulating the learning process of two agents that acquire a shared signalling system in a simple setup with a one-dimensional world and a one-dimensional form space, as illustrated in Fig. 2. Our experiments provide two essential insights: First, the optimal solution with a continuous mapping (Fig. 2(a)) is not always found and the chances of finding it are highly dependant on external parameters, such as the noise in form transmission. Second, the sub-optimal solutions (Fig. 2(b)) with a partially discrete and fragmented signalling convention are *locally* optimal, that is, once being established they are stable and highly unlike to change.

#### 2.1.3 Topological mismatch

The second reason for the emergence of discretisation in an entirely continuous setting is a more fundamental one: There are certain situations, in which a continuous mapping from forms to meanings is impossible, even though both spaces are perfectly continuous. This is the case if the space of forms and the space of meanings have a different topology.

**Fig. 3:**
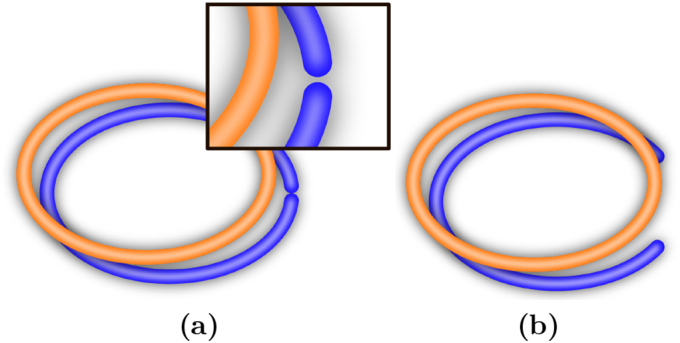
Topological mismatch between form space (orange) and meaning space (blue). (a): Misunderstandings occur because the boundaries of meaning space are mapped close to the same point in form space (inset). (b): A gap in form space avoids misunderstandings. (For interpretation of the references to colour in this figure legend, the reader is referred to the web version of this article.)

In our experiments, we use the simple example of a circular form space and a bounded line as meaning space, as illustrated in Fig. 3. The form space can be continuously mapped onto the meaning space, except for the boundaries of the meaning space. At this point, two maximally different meanings are mapped next to each other in form space. To avoid misunderstandings in case of noisy form transmission, the boundaries of the meaning space have to be mapped at a certain distance, leaving a gap in form space. Forms within this gap region cannot be used to reliably communicate meaning. This is the simplest case of a discretisation that emerges due to a topological mismatch between form and meaning space.

In reality, the relevant topologies are of course much more complex. For instance, the human vowel system has at least three major dimensions (tongue position, tongue height, lip roundedness) and several subordinate dimensions that are not generally independent (Ladefoged & Maddieson, 1990). Beyond language, music has the capacity to convey emotions and other complex states of mind (Koelsch, 2013; Meyer, 1956). The spaces of musical objects, such as tones, chords or interacting polyphonic voices, have a highly complex topology. For instance, the space of musical keys and triads alone has a topology that can be alternatively described as a planar two-dimensional *Tonnetz* (Euler, 1739; Riemann, 1896), a tube (by rolling up the Tonnetz), or a torus (by additionally rolling up the tube). Which of these topologies is most appropriate to describe music perception depends on which properties of the notes are considered to be relevant: The distinction between Pythagorean and syntonic major thirds is dropped in the first step; enharmonic differences in the second; and octave equivalence is assumed in all three topologies (Chew, 2000; Cohn, 1997; Krumhansl, 1998; Krumhansl & Kessler, 1982; Lieck, Moss, & Rohrmeier, 2020; Milne & Holland, 2016). This kind of cyclic topology in form space allows, for instance, to compose musical sequences that induce the impression to constantly rise ad infinitum, such as the Shepard tone illusion (Shepard, 1964).

These are only two specific examples of non-trivial topologies that arise in the real world. There are many more modes of communication, each with their own topological particularities. It is therefore highly plausible that the topological effects we observe in our experiments also play a role in real-world communication.

### 2.2 A unified description of discrete and continuous properties

Our second major concern in this paper is to propose a unified description of the discrete and continuous properties of signalling systems.

There are accurate and mathematically concise descriptions of both discrete and continuous spaces. Moreover, many theories (including probability and information theory) can be formulated on an abstract level and are applicable to discrete and continuous spaces without having to change the formalism. However, a central problem in the description of real-world signalling systems is that, in a way, they are discrete and continuous *at the same time*: Depending on the level of description, a digital computer can be understood to operate on binary Boolean values or on the basis of continuous voltages. We believe that mistaking a difference in the level of description for the fundamental question of whether something is “truly” discrete or continuous is one of the most common reasons for confusion.

To allow for a productive discussion about the relation between discretisation and continuity in communication, it is therefore important to bridge this gap between the different levels of description. A crucial step forward in that respect is the approach by Feldman (2012) to identify conditions on which a continuous world can be adequately described using a discrete representation.

In this paper, we expand on this idea: Our goal is to describe communication in a way that allows for discrete and continuous properties to coexist as part of the *same* representation. This is indispensable to describe the emergence of discretisation, as it has to be identified in hindsight without introducing it beforehand. We therefore start off with a continuous representation and provide a definition for discrete entities on its basis.

**Fig. 4:**
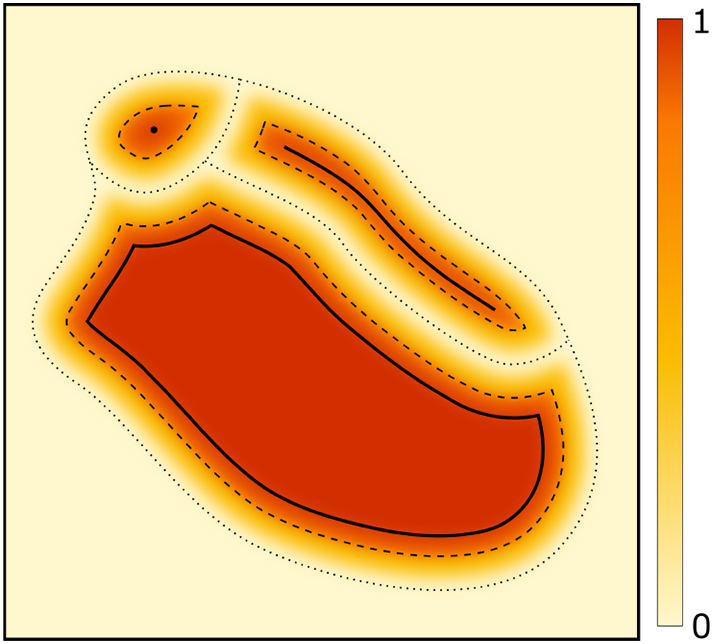
A hypothetical form space with three symbols. The *colour scale* indicates how likely it is for a specific form (i.e. a point in the space) to occur in communication. Forms outside the regions indicated by *dotted lines* effectively do not occur (e.g. because they are practically impossible to produce); dotted lines also indicate the decision boundary between symbols. *Dashed lines* indicate regions that are typical for a specific symbol; these forms can safely be used in communication without a considerable risk of misunderstandings, even in the presence of noise. *Solid lines* indicate idealised forms of a symbol; this is the symbol's internal form space with an iconic mapping, where continuous changes of the form induce analogous changes in meaning. For two of the symbols, the internal (iconic) space is of non-degenerate dimension one and two, respectively, while the top-left symbol is a purely discrete (atomic) symbol with a degenerate internal space of dimension zero.

Fig. 4 gives a general intuition of our conception of a unified discrete-continuous representation. The formal definitions and mathematical details can be found in Section 5.1.

## 3 Related work

### 3.1 Communication games

In the last two decades, the synthetic approach to language evolution has become technically tractable (Nolfi & Mirolli, 2010; Steels, 1997). In particular, communication games (Steels, 1997, Steels, 1998; Wittgenstein, 1953) have been used to study various properties of language and language evolution via robotic experiments and computer simulations of communicating agents (Brighton & Kirby, 2006; Cangelosi & Parisi, 2002; Christiansen & Kirby, 2003; Oliphant & Batali, 1997; Van Eecke & Beuls, 2020).

In the standard setup for communication games, two or more agents are equipped with a predefined set of discrete symbols.^3^ The sender and the receiver repeatedly engage in communication by exchanging a symbol or a combination of symbols and receive feedback about the quality of their communication. Depending on the specific setup, the symbols are either transmitted as discrete units or transformed into a continuous representation, such as sound, by the sending agent, which is then mapped back to a discrete symbol by the receiving agent. Existing research has mainly focused on one of three distinct tasks.

#### 3.1.1 Syntax and semantics

The majority of works is concerned with the question how the rules for combining symbols (syntax) can be learned and how the resulting symbol combinations acquire meaning (semantics) (Bleys, Loetzsch, Spranger, & Steels, 2009; De Jong, 1999; Nowak & Krakauer, 1999; Oudeyer & Kaplan, 2007; Spranger, 2016; Steels, 1997, Steels, 1998; Steels & Belpaeme, 2005). In this case, the agents have to learn how to choose and combine symbols based on their perception of the world (sender) and how to interpret the received symbols correctly (receiver). The quality of the communication is measured based on whether the intended features of the world were successfully transmitted from sender to receiver. These approaches conceptually stay within the purely discrete realm.

#### 3.1.2 Vocalisation

A smaller part of the field is concerned with learning the mapping from discrete symbols to a continuous signal that is transmitted between the agents. This does not necessarily involve the actual communication of content, but the goal of the agents rather is to imitate each other as accurately as possible (imitation games, Steels, 1998) to ensure the correct transmission of the actual discrete symbols. For instance, it has been investigated how a vowel system develops by modelling the actual articulation and perception of sounds (de Boer, 2000; Oudeyer, 2005) and how agents learn to vocalise relevant phonetic units (Moulin-Frier, Nguyen, & Oudeyer, 2014; Moulin-Frier & Oudeyer, 2012; Murakami, Kröger, Birkholz, & Triesch, 2015).

#### 3.1.3 Emotional speech synthesis

The third task is the expression of emotions in speech synthesis by shaping the continuous representation of the discrete symbols appropriately. Communication games are one approach how agents may learn to convey emotions by mapping them to prosodic features, such as pitch and duration (Oudeyer, 2003). Other techniques for emotional speech synthesis date back several decades (Schröder, 2001) and the underlying principles are shared with other forms of non-vocal communication such as music (Juslin & Laukka, 2003). However, while these approaches address the continuous properties of symbols, they are not concerned with the emergence of the symbols themselves.

### 3.2 Discretisation of forms

A discretisation of the continuous vowel space into distinct vowels prototypes is assumed by de Boer (2000). The number of prototypes as well as their exact form (their actual sound defined by their location in vowel space) is learned. In our terms, each of these vowels prototypes essentially corresponds to an atomic symbol with a single idealised form. However, these symbols did not emerge from a continuous space but were injected as predefined discrete units.

Oudeyer (2005) suggests a similar approach, where a single act of communication consists of a trajectory through vowel space that interpolates between a small number of randomly selected vowel prototypes. The set of all prototypes is pre-populated with a large number of random prototypes, whose form is updated after each communication. Due to the update operation, which effectively moves nearby prototypes closer together, they cluster in vowel space. These clusters take a similar role as the distinct prototypes in (de Boer, 2000) without the need to explicitly represent their number.

Furthermore, Vallabha, McClelland, Pons, Werker, and Amano (2007) suggest an unsupervised clustering method for learning categories in vowel space using data of infant-directed speech.

These works are interesting in that they suggest possible mechanisms how a discrete structure could have emerged in form space, independently of any meaning that is to be communicated. However, precisely for that reason they do not allow to investigate the interplay between discretisation and continuity in communication, which is the central goal in this paper.

### 3.3 Form-meaning mappings

The closest precursor to our work is the one by Zuidema and Westermann (2003). While working in an entirely discrete setup, they introduce a continuous topology in form and meaning space by means of a noisy transmission distribution and a reward/value function. Our sender policy, receiver policy, transmission distribution, and reward function are the equivalent of their *S*, *R*, *U*, and *V* matrices. Due to working in a discrete setting, they can compute the transmission distribution for *meanings* (*p*(*m*′| *m*); our (12)) in closed form to directly maximise the expected reward (minimise the distortion). Their experiments are similar to our non-cyclic one-dimensional case and they also observe fragmented form-meaning mappings for little noise and smoother mappings for an increased noise level. Likewise, they conclude that in an optimal signalling convention the sender and receiver policies should be unambiguous (which they call specificity and distinctiveness) and the topology of the form and meaning space should be preserved (regularity).

Our experiments generalise their setup in several respects. First, we use truly continuous form and meaning spaces. The sender and receiver policy therefore have to be represented by continuous functions instead of matrices and all sums over forms and meanings have to be replaced by integrals (their circle plots are the discrete equivalent of our heat maps). Second, we only use the reward feedback of single communication acts for learning. They present preliminary results for this scenario with “limited feedback” but do not observe a preservation of topology, as we do in our experiments. Third, we investigate the effect of different and more complex topologies and, in particular, the effect of a topological mismatch.

Finally, we provide a theoretical basis for understanding the observed effects and investigating them empirically by 1) providing a unified mathematical description for discrete and continuous properties and 2) drawing the connection to information theory.

### 3.4 Continuous mappings and topology

The interplay of continuous (iconic) mappings and discretisation in the case of a topological mismatch between the form and meaning space was empirically investigated in humans by Little, Eryılmaz, and de Boer (2017). In theory and simulations, the case of a topological mismatch is addressed by de Boer (2012) and de Boer and Verhoef (2012). They note that a continuous (iconic) mapping is not possible in that case but restrict their considerations to the case where the form space has a lower dimensionality than the meaning space (1D versus 2D in their experiments). They conclude that in this situation a discretisation in *meaning* space must occur but do not formalise this notion. In our terms, what they mean is a benign ambiguity of the sender policy, which is forced by the topological mismatch. This does not imply a discretisation in form space (as is required for the emergence of symbols) since all available forms are equally used to convey a specific meaning.

Ellison (2013) attempts to extract topographic mappings from form-meaning samples in a 1D-1D-setting, comparing random with highly correlated mappings. While he does not explicitly consider communication between agents and does not include any learning component, we can still draw a connection to our findings on local optima with a fragmented form space: For samples from a random form-meaning mapping, which is the initial state of our learning process, he finds that the best topographic mapping is highly fragmented. In our experiments, we explicitly counteract fragmentation by means of transmission noise and exploratory policies, but we observed similar results for very low values of transmission noise and exploration. Conversely, the optimal policies learned by our agents correspond to his case of a highly correlated from-meaning mapping with non-fragmented form space.

We expand on the existing studies by performing experiments with more complex topologies, in particular ones that induce a discretisation in form space, and providing a formal basis for describing and interpreting the results.

## 4 Experimental method

We use a similar setup as in previous works based on simulated communication games (esp. Zuidema & Westermann, 2003; de Boer & Verhoef, 2012), with the important difference that we generalise to the fully continuous case. That is, we do not assume the existence of discrete symbols or categories at any point and all steps operate in continuous form and meaning space. Discretisation is thus not a prioi built into our setup, instead, we show that discrete symbols embedded into these continuous spaces emerge as a result of the learning process.

### 4.1 Communication process

**Fig. 5:**
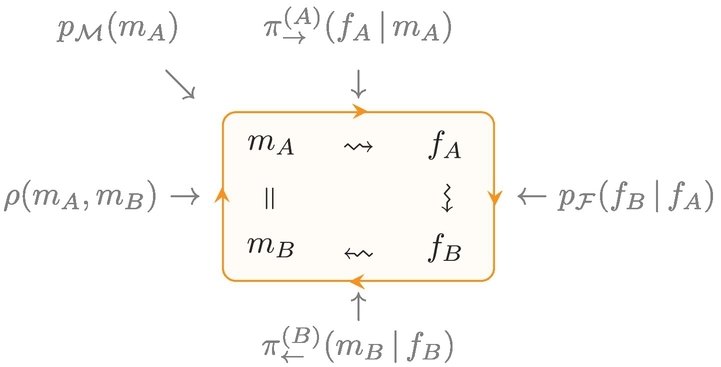
Communication cycle from *A* (sender) to *B* (receiver): a meaning *m*_*A*_ ∈ ℳ corresponding to *A*’s perception of the world is sampled from the meaning distribution *p*_ℳ_; *A* chooses a form *f*_*A*_ ∈ ℱ to communicate *m*_*A*_ according to its sender policy *π*_→_^(*A*)^; this form is transmitted as *f*_*B*_ ∈ ℱ to *B* via the transmission distribution *p*_ℱ_; *B* interprets the form *f*_*B*_ to have the meaning *m*_*B*_ ∈ ℳ according to its receiver policy *π*_←_^(*B*)^; finally the success of communication is evaluated by comparing *m*_*A*_ and *m*_*B*_ and both agents receive a reward according to the reward function *ρ*.

Two agents, *A* and *B*, live in a world with continuous meaning space ℳ and exchange forms from a continuous form space ℱ. They repeatedly engage in communication, where a single act of communication from *A* to *B* consists of: *A* perceiving the world as *m*_*A*_; choosing a form *f*_*A*_ for communication; *f*_*A*_ being transmitted to *B* as *f*_*B*_, *B* interpreting the form as *m*_*B*_, and both agents receiving a reward depending on how close the original meaning *m*_*A*_ perceived by *A* and *B*’s interpretation *m*_*B*_ of the received form are. This process is depicted in Fig. 5 and the separate steps are now described in more detail.

The form and meaning space correspond to the unit cubes ℱ = [0, 1]^*d*_*f*_^ and ℳ = [0, 1]^*d*_*m*_^ of dimensionalities *d*_*f*_ and *d*_*m*_, respectively. Additionally, any dimension may have cyclic boundary conditions by gluing together the corresponding faces. The sender's perception of the world (i.e. the meaning that is to be communicated) is sampled from the meaning distribution *p*_ℳ_.^4^ More complex topologies than the unit cube (such as the *TwoLines* world model used below) can be defined by choosing an appropriate reparameterisation and/or meaning distribution.

The sending agent chooses a form according to its sender policy *π*_→_(*f*| *m*); the receiving agent interprets the form according to its receiver policy *π*_←_(*m*′| *f*′). These policies are learned by the agents through interaction, as described in Section 4.2.

Forms are transmitted from the sender to the receiver with additional noise defined by the transmission distribution *p*_ℱ_(*f*′| *f*). Transmission noise is either Gaussian with mean at *f* and standard deviation *σ* or corresponds to a mixture of two Gaussians with mean at *f*, standard deviations *σ*_1_ and *σ*_2_ and mixture weights *α*_1_ and *α*_2_, respectively. This second version allows us to add heavy-tailed noise to an otherwise narrow transmission distribution when investigating convergence properties.

The communication success is measured by the reward function *ρ*(*m*, *m*′), which is a Gaussian with standard deviation *σ*_*ρ*_: A maximum reward is achieved if the sender's perception of the world (the intended meaning *m*) and the interpretation *m*′ of the receiver are identical. We use a value of *σ*_*ρ*_ = 0.2 in all our experiments. After each communication act, the resulting reward is observed by both agents. This is the only kind of feedback they obtain to improve their communication policies. In particular, each agent only has access to their own form and meaning not to those of the other agent.

**Fig. 6:**
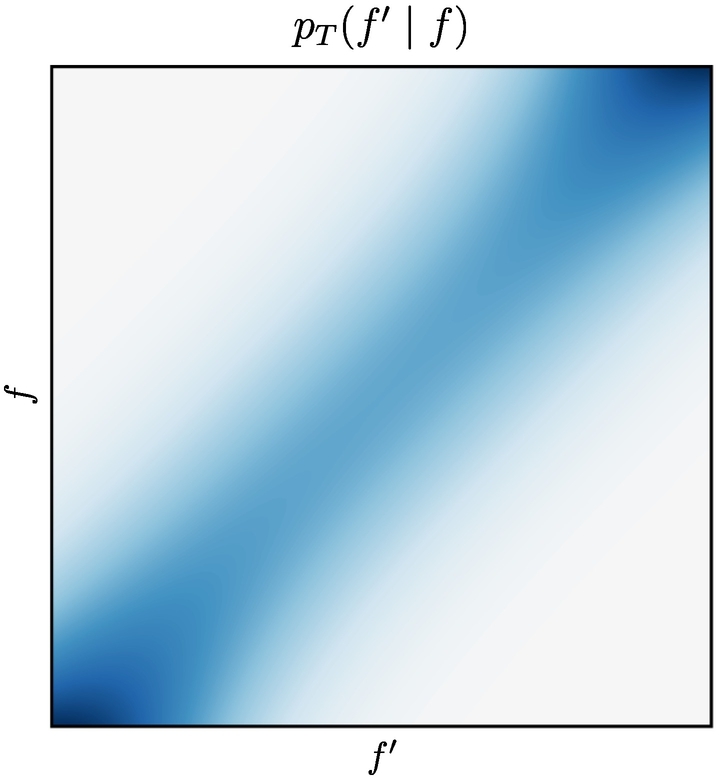
Effective transmission distribution with renormalisation if *f* is near the boundaries, leading to a lower entropy and variance in these regions (boundary effect).

We ensure forms and meanings to remain inside the unit cubes in the presence of noise by either wrapping them around (performing a modulo operation) in case of cyclic boundary conditions or by rejecting communication acts with out-of-bound values.^5^ This corresponds to implicitly truncating and renormalising the transmission distribution near the boundaries, which results in lower noise (a lower conditional entropy and variance) in these regions. The effective transmission distribution is shown in Fig. 6. This *boundary effect* can be observed in our simulations as the agents develop a slight preference for these regions in form space due to the reduced transmission noise (also see Section 7.2.2).

### 4.2 Learning

The agents attempt to maximise the reward they expect to receive after each act of communication by adapting their sender and receiver policies. In our setup, the only information they have are their own form and meaning and the reward that both agents receive. For each communication act they remember the triplet (*f*, *m*, *ρ*) consisting of the form *f*, the meaning *m*, and the received reward *ρ*. While collecting this data, they do not differentiate between whether they were sender or receiver, that is, whether they chose form *f* to communicate meaning *m* (their perception of the world) or whether they chose *m* as the interpretation of the received form *f*.

This implies a coupling of each agent's sender and receiver policy, making them consistent for each agent individually (but not necessarily between agents). Such a coupling of production and perception of forms is cognitively plausible and has been used before (de Boer, 2000; Oudeyer, 2005). In a two-agent setup it also prevents a situation where one agent speaks language A and understands language B, while the other speaks B and understands A, a problem that does not exist with a population of three or more agents.^6^

Learning good sender and receiver policies only from rewards is challenging because the rewards themselves depend on the policies. This is known as the reinforcement learning problem (Sutton & Barto, 2018) and can be solved using an iterative procedure called *policy iteration* (Lagoudakis & Parr, 2003). Starting with a random initial policy, an agent iterates three steps:

1. collect data using the current policy
2. use this data to estimate the expected reward for each form-meaning pair
3. update the policies to maximise the expected reward.

This strategy is guaranteed to converge to a locally optimal policy as long as the agents perform a minimal amount of *exploration*, which means that they should from time to time also choose actions that seem sub-optimal. Intuitively, this ensures that they do not miss out on good alternative policies. The trade-off between choosing optimal actions and exploring alternative options is known as *exploration exploitation dilemma*.^7^

To collect data, the agents perform 5000 acts of communication in each direction. Each of these 10,000 data points specifies the reward *ρ* that was obtained at a point (*f*, *m*) in form-meaning space. From these data, each agent now has to estimate the expected reward and then update its policy.

#### 4.2.1 Estimating the expected reward

Technically, estimating the expected reward from these interaction data is a supervised learning problem, specifically, a regression problem (Bishop, 2007; Hastie, Tibshirani, & Friedman, 2008). This means that the reward *ρ* at each point (*f*, *m*) is understood to be a noisy observation of some underlying function *r*(*f*, *m*). A common approach to finding this function is to:

1. assume *r*(*f*, *m*) to be of a specific mathematical form with a number of free parameters that can be adjusted
2. define an objective or loss function that measures how well *r*(*f*, *m*) with a specific set of parameters matches the data
3. adjust the parameters to achieve the best fit (minimal loss).

We are taking a standard approach, known as *generalised linear regression*, and assume *r*(*f*, *m*) to be a linear combination of a set of basis functions

(1) $$r\left(f,m\right)=\sum_{i}\beta_{i}\;b_{i}\left(f,m\right),$$

where *b*_*i*_ are the individual basis functions and *β*_*i*_ are the open parameters that are to be adjusted. To define the loss function, we assume the observed rewards *ρ* to be normal distributed around the true function *r*(*f*, *m*), which is equivalent to using the *mean squared error* as the loss function for evaluating the parameters *β*_*i*_. Furthermore, we add an *L*_2_-regularisation of strength 1 to the parameters, which increases numerical stability and counteracts overfitting. Intuitively, this regularisation pushes the parameters *β*_*i*_ towards small values, which results in single outliers having a smaller effect and regions without any data being estimated to have zero reward. Using this approach, the optimal parameters can be found efficiently and in closed form (Bishop, 2007; Hastie et al., 2008).

The basis functions *b*_*i*_ determine what shapes *r*(*f*, *m*) can possibly take. In our case, the primary interest is to obtain a relatively smooth function for two reasons. The first is a technical one: Estimating a smooth function is more robust against noise, especially if the number of observations is small. The second and more important reason is cognitively motivated. Even though we are abstracting from the specific biological (or technical) characteristics of the agents, the core elements that determine the outcome of our experiments should still be cognitively plausible. A smooth function that reflects proximity relations in the form and meaning space is much more likely to be found in a biological system.

We therefore choose our basis functions to be Gaussians that are placed on an equidistant grid and have a standard deviation equal to the grid spacing (see Appendix A for an illustration). This arrangement ensures that *r*(*f*, *m*) has a smooth shape and (due to the large overlap between basis functions) the underlying grid does not manifest itself in the estimate of the expected reward.

#### 4.2.2 Policies

Given an estimate of the expected reward *r*(*f*, *m*), the agents have to update their sender and receiver policies, *π*_→_(*f*| *m*) and *π*_←_(*m*| *f*). Generally, the updated policies should maximise the expected reward. However, as mentioned above, for a robust learning process it is also important to perform a certain amount of exploration by taking seemingly sub-optimal actions in order discover better policies in the next iteration. We therefore have different options for updating the policies.

The *optimal* policy^8^ would deterministically choose the form or meaning with the maximal expected reward, *argmax r*(*f*, *m*), without performing any exploration at all. An *ε*-*optimal* policy chooses uniformly among the forms or meanings with near-optimal reward, where *ε* regulates the tolerance:^9^ *ε* = 0 corresponds to the optimal policy and *ε* = 1 chooses among all forms or meanings, ignoring the expected reward. Exploration is therefore either restricted to a near-optimal region (for small *ε*) or the expected reward is largely ignored (for large *ε*). The optimal and *ε*-optimal policies are interesting for theoretical considerations and we will get back to them later. But they are not well suited for learning because they do not balance exploration and exploitation well.

Therefore, during learning, the policies are updated to choose forms and meanings with a probability proportional to the expected reward

(2) $$\pi_{\to }\left(f|m\right)=\frac{r\left(f,m\right)}{\int_{ℱ}r\left(f^{\prime },m\right)df^{\prime }}$$

(3) $$\pi_{\leftarrow }\left(m|f\right)=\frac{r\left(f,m\right)}{\int_{ℳ}r\left(f,m^{\prime }\right)dm^{\prime }},$$

where the integrals in the denominators are for normalisation. This choice assigns the highest probabilities to the best forms and meanings but at the same time performs exploration in all regions with non-zero expected reward. The specific representation of the expected reward function as a linear combination of Gaussians allows us to efficiently sample from these policies (see Appendix A for details).

Note that *π*_→_(*f*| *m*) and *π*_←_(*m*| *f*) correspond to the send and receiver matrices in (de Boer & Verhoef, 2012; Zuidema & Westermann, 2003) and in a discrete setting, the integrals in (2) and (3) would need to be replaced by sums over forms and meanings, respectively.

## 5 Discrete symbols in continuous spaces

Explaining the emergence of symbols in an entirely continuous setting requires a rigorous definition of what discrete symbols are in this case and how they are embedded in the continuous space. We provide such a definition below and also describe how it can be practically evaluated on experimental data, which allows us to detect the emergence of symbols in our simulations.

Before we come to the technical details, we would like to motivate our general conception. An illustration of how we define discrete symbols in a continuous space was already given in Fig. 4. Take our example with the word “very” in Section 1.3. This word can be considered a prototypical example of what is commonly called a discrete symbol: It is listed in a dictionary and if it occurs multiple times, all of these occurrences are generally though of as being the *same* word and conveying the *same* meaning (even though context may have an influence). However, in our example, we have tried to demonstrate that pronouncing or printing this word differently may in fact induce slight changes in its meaning – changes that can be intentionally controlled to shape its exact meaning in a specific way. When zooming in on these slight changes, the word “very” does not seem to be perfectly discrete anymore, but instead it seems to contain a small meaning space within, which is, in a way, encapsulated inside the discrete symbol.

Starting from this observation, we would like to define a *symbol* in a way that preserves both its discrete character and its internal meaning space. Put differently, we still want to be able to speak of “very” and “different” as two distinct words (symbols), but at the same time we want to be able to speak about their continuous variations in meaning if being pronounced differently.

Furthermore, what a symbol is cannot only be defined in terms of forms, but we also need to take meaning into account. In fact, it is perfectly possible to produce all kinds of sounds that could potentially be used to convey meaning (and maybe they even are in a culture different from ours), but they are gibberish to our ears, their form is not associated to any symbol, and they do not properly convey any meaning (to *us*). Our definition therefore has to be built upon the actual signalling conventions that have been established by a specific group of agents in a specific world. This brings us to defining:

*A symbol is a connected region in form space, in which all forms can be effectively used in communication and that is separated from other symbols.*

The form component enters this definition quite explicitly, while the meaning component is introduced through the actual usage in communication. The general idea of discrete elements being represented as delimited regions in a continuous embedding space is somewhat similar to that of *conceptual spaces* (Gärdenfors, 2004), but our notion of symbols is concretely specified in terms of the expected reward and relies on fewer assumptions (e.g. we do not require convexity). We will now formulate this definition more precisely in mathematical terms.

### 5.1 Definition of symbols

First, we need to define what “can be effectively used in communication” means. One approach is to consider whether a form effectively *is* used in communication by inspecting a concrete sender policy or communication data collected with it. This is an appropriate fallback option if we do not have access to the agent's internals or the world model. However, we would like to sharpen our general definition by considering whether a form *can* be used, that is, whether it is generally *permissible* for communication. We therefore need to define a criterion for permissibility that is independent of the agent's actual policy.

#### 5.1.1 Permissibility

The basic idea of *permissibility* builds on the concept of *ε*-optimal policies mentioned in Section 4.2.2. An *ε*-optimal policy chooses among the best *ε*-quantile of forms to convey a specific meaning. Accordingly, we define a form to be *ε*-permissible for communicating a specific meaning if it can be used by an *ε*-optimal policy. To decide whether a form *f* is *ε*-permissible for a specific meaning *m*, we have to compare its expected reward *r*(*f*, *m*) to that of all other forms *f*′ and determine whether *f* is within the top *ε*-quantile. Formally, we have:

Definition 1 (Permissibility). A form *f* ∈ ℱ is *ε*-permissible for communicating meaning *m* ∈ ℳ, written *f_ε_*⊨*m*, if and only if

(4) $$\frac{1}{|ℱ|}\int_{ℱ}〚r\left(f^{\prime },m\right)>r(f,m)〛df^{\prime }\leq \varepsilon ,$$

with 0 ≤ *ε* ≤ 1, and where 〚⋅〛 is the Iverson bracket, which is 1 if the contained expression is true and 0 otherwise.

The left-hand side of (4) may become more intuitive if we think about its discrete analogue: In the discrete case, it simply counts the number of forms *f*′ that have a higher expected reward than *f* for the meaning *m*; it then divides this number by the number of all forms. The left-hand side of (4) thus computes the relative volume of all forms that are better than *f* for communicating *m*. If this relative volume is equal or smaller than *ε*, then *f* is *ε*-permissible for communicating *m*.

We are using the notation *f*_*ε*_⊨*m* in loose analogy to how it is used to denote logical consequence: *f*_*ε*_⊨*m* can be thought of to mean that observing the form *f* (logically) entails that the meaning *m* was intended to be communicated. The value of *ε* determines how narrow the bounds for permissibility are chosen. In the limit *ε* = 0, the set of *ε*-permissible forms shrinks down to contain only the single form that is best for communicating *m*.

#### 5.1.2 Symbols

The definition of permissibility depends on a specific meaning *m* that is considered: *f* is only ever permissible (or not permissible) for a specific *m*. This may include highly unusual meanings that are best communicated using highly unusual forms. It is, for instance, conceivable that in a very unusual situation, a person is entirely overwhelmed and, trying to somehow express her state of mind, utters some gibberish. And it may be that for a second person, it is in this precise situation exactly this gibberish that most accurately conveys this meaning (i.e. the first person's state of mind). We are not arguing that this is very likely to happen, only that it may in principle come about and therefore has a probability greater than zero (even if vanishingly small). To arrive at a definition of whether a form *f* is permissible *in general*, we therefore have to take the meaning distribution *p*_ℳ_ into account.

The probability *p_ε_*_⊨_(*f*) of a form *f* to be *ε*-permissible in communication given the meaning distribution *p*_ℳ_ is

(5) $$p_{\varepsilon ⊨}\left(f\right)=\int 〚f_{\varepsilon }⊨m〛p_{M}\left(m\right)\;\mathrm{dm}.$$

This is equivalent to the probability of a form to be used in an *ε*-optimal policy. To decide whether *f* is *generally* permissible, we again have to decide on a tolerance level. We say that *f* is (generally) *ε*-permissible if *p*_*ε*__⊨_ (*f*) > *δ* for some small value 0 ≤ *δ* ≤ 1.

We now have a clear definition of what it means to say that a form “can be effectively used in communication” and can move on to defining what a symbol is.

Definition 2 (Symbol). A symbol f⊆ℱ is a maximal connected region in form space, in which any form *f* has a probability of being *ε*-permissible *p*_*ε*__⊨_(*f*) larger than *δ* ≥ 0

(6) $$\forall f\in f:p_{\varepsilon ⊨}\left(f\right)>\delta ,$$

where connected means that between any two forms of the symbol there is a path that lies entirely within the symbol and maximal means that no form can be added to the symbol without it ceasing to be one.

The requirement to be *connected* ensures that we cannot add arbitrary forms to a symbol but only those that create a unit with the existing ones. The requirement to be *maximal* implies that different symbols have to be separated: If they were not, a path would exist between them so that we would need to extend one into the other and they would become one.

This definition has advantageous properties from both a theoretical as well as a practical point of view. The two parameters *ε* and *δ* allow for an adaptation to the specific circumstances. For theoretical considerations we can take the limit *ε* = 0 and *δ* = 0, while for practical investigations values larger than zero will generally be beneficial.

*ε* determines to what extend sub-optimal communication is taken into account: A value of *ε* = 0 means that for any meaning, we only consider the best form, that is, we ignore any mistakes or inaccuracies that may occur in real-world communication. Many forms that are near-optimal will then have a zero probability *p*_*ε*__⊨_ of being permissible and will thus not be part of any symbol. In contrast, small non-zero values of *ε* will include these near-optimal forms, which may be used in practical applications to increase robustness against noise. Generally, *ε* can be understood as a kind of smoothing parameter in form space. Obviously, values of *ε* that are too large will include many forms that cannot reasonably be used in communication, which will obfuscate the structure of the form space and make the detection of symbol boundaries impossible. In the hypothetical example in Fig. 4, the solid lines correspond to symbol boundaries with a value of *ε* = 0, the dashed lines correspond to a small non-zero value of *ε*, while the dotted lines are on the limit of blurring the symbol boundaries. The heat map would change depending on the value of epsilon, with non-zero values only within the symbol boundaries (assuming a value of *δ* = 0). The shown heat map corresponds the value for the dotted lines.

*δ* is related to the meaning distribution *p*_ℳ_. A value of *δ* = 0 is useful for worlds in which there is a clear distinction between important meanings that have a non-zero probability *p*_ℳ_ > 0 and unimportant meanings with a probability *p*_ℳ_ = 0 that is strictly zero. If the meaning space may have elements that should be ignored, even though their probability *p*_ℳ_ is not strictly zero, we can choose a value *δ* > 0 greater than zero to ignore them. Note, however, that *δ* is applied to the probability *p*_*ε*__⊨_ of the forms to be permissible, which means that many low-probability meanings may theoretically accumulate in a single form, which then has a significantly higher probability of being permissible. Applying *δ* to *p*_*ε*__⊨_ instead of *p*_ℳ_ is an intentional choice because the analytical value of *p*_ℳ_ may not be know in some applications, whereas *p*_*ε*__⊨_ is amenable to approximations, as discussed in the next section.

### 5.2 Detecting symbols empirically

If we want to detect symbols in empirical investigations, we need a way to evaluate the relevant condition (6) in Definition 2. In principle, this requires us to know the meaning distribution *p*_ℳ_ in (5) and the expected reward *r*(*f*, *m*) in (4), both of which may not be readily available. Moreover, even if they are available, the integrals may be intractable to solve analytically.

Fortunately, both equations are amenable to approximations based on Monte-Carlo sampling (Robert & Casella, 2004). The basic idea is that instead of integrating over all possible values, we take the average of the integrand for a finite number of randomly drawn samples. The result will be close to the true value, if the number of samples is large. In (4), the samples are distributed uniformly, while in (5) they are drawn from *p*_ℳ_, which means that we do not need to know the value of *p*_ℳ_ as long as we can sample from it. The details are described in Appendix B.

We can thus approximate *p*_*ε*__⊨_ as long as we can evaluate the expected reward *r*(*f*, *m*) and sample from *p*_ℳ_, which is the case in our experiments. In cases where either of both is not possible, but communication data are available, *p*_*ε*__⊨_ can be replace with the probability of a form being de facto used in communication (estimated by its relative frequency), and this value can be used to evaluate the condition (6) in Definition 2.

#### 5.2.1 Permissibility analysis

To identify symbols, we need to do a *permissibility analysis*. This corresponds to performing the above computations, step by step, and interpreting the results. Additionally, considering different values for *ε* and *δ* as part of the analysis is usually helpful. We will take an example from our experiments to explain the separate steps in detail. The result of the permissibility analysis is shown in Figue 7 and corresponds to the fragmented one-dimensional scenario that was illustrated in Fig. 2(b).

**Fig. 7:**
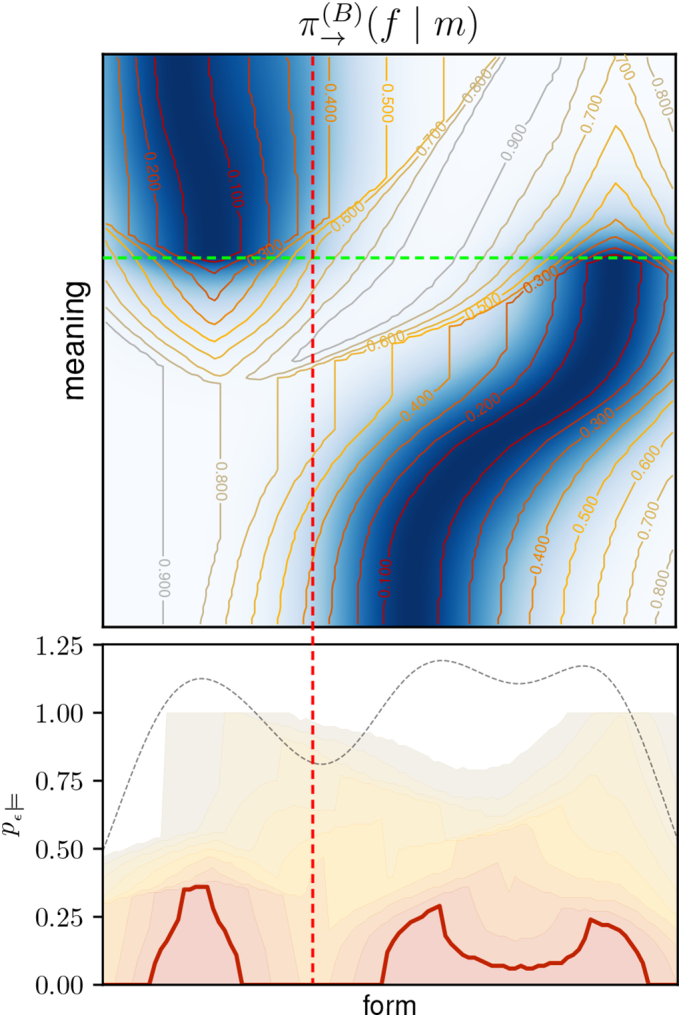
Permissibility analysis of example from Fig. 9 (see text for details).

The first step is to evaluate the expected reward *r*(*f*, *m*) for every form-meaning pair in order to find the best forms for each meaning. The upper plot in Fig. 7 shows the sender policy as a heat map, which corresponds to the normalised expected reward.^10^ Second, we need to choose a value for *ε* and find for each meaning all *ε*-permissible forms. This is indicated by the contour lines in the upper plot, using different values for *ε*, and allows for analysing the permissibility relations between different forms and meanings:

Horizontal lines (slices along the form space) can be used to analyse which forms are permissible for a specific meaning: Forms within the *ε*-permissible contour lines (or hyper-surfaces in higher dimensions) are permissible for the respective meaning. For instance, the fact that the dashed green line intersects with the *ε*-permissible region for *ε* = 0.1 at *two* locations indicates that two very different forms are 0.1-permissible for this specific meaning – a consequence of the fragmented form space, as we will discuss later.

Vertical lines (slices along the meaning space) show what meanings a particular form is permissible for: The form is permissible for meanings within the *ε*-permissible contour lines (or hyper-surfaces).

To compute the probability *p*_*ε*__⊨_(*f*) of a specific form *f* to be *ε*-permissible, we need to integrate over all meanings for which *f* is permissible. During integration, meanings need to be weighted with their probability *p*_ℳ_(*m*) to occur. In this specific example, the meaning distribution is uniform so that *p*_*ε*__⊨_(*f*) simply corresponds to the percentage of meanings for which *f* is *ε*-permissible. For instance, the vertical dashed red line intersects only with *ε*-permissible regions for *ε* > 0.3 in the upper plot. The probability *p*_*ε*__⊨_ of the corresponding form is therefore zero for all values *ε* ≤ 0.3 in the lower plot.

The corresponding values are indicated in the lower plot in Fig. 7 (for the same values of *ε*) and correspond to the probability of a form to be used in an *ε*-optimal policy. To improve clarity, we do not repeat the labels and use a thicker line for *ε* = 0.1, as it corresponds to the most relevant case of a near-optimal policy. For additional information, the plot also shows the probability *π*_→_(*f*) of a form to be used by the agent's actual sender policy (dashed grey line). We see that *π*_→_(*f*) basically corresponds to a smoothed version of the near-optimal policy, which is due to its exploratory character that is required for robust learning.

We can now identify the existing symbols by choosing a value for *ε* and *δ* and looking for connected regions in form space for which *p*_*ε*__⊨_ is greater than *δ*. In this case, choosing *δ* = 0 and *ε* = 0.1 (thick red line), we find two distinct symbols that persist for all values up to *ε* = 0.3.

This kind of permissibility analysis will be crucial for interpreting our experimental results presented in the next section.

## 6 Experiments

Our experiments comprise four main parts. First, we will introduce the general setup in a simple 1D-1D-setting, explain the employed visualisations, show a typical learning progress, and discuss some general properties of the learned signalling conventions. Second, we investigate convergence properties in the same 1D-1D-setup by analysing an example of sub-optimal signalling conventions with a fragmented form space and performing an extensive statistical analysis for different noise distributions. Third, we investigate the scenario of modal worlds and show that, indeed, the modal structure is reflected in distinct symbols. Finally, we perform a simulation with a mismatch in the topology of the form and meaning space, showing how this leads to a discretisation in form space.

### 6.1 1D-1D-setting

**Fig. 8:**
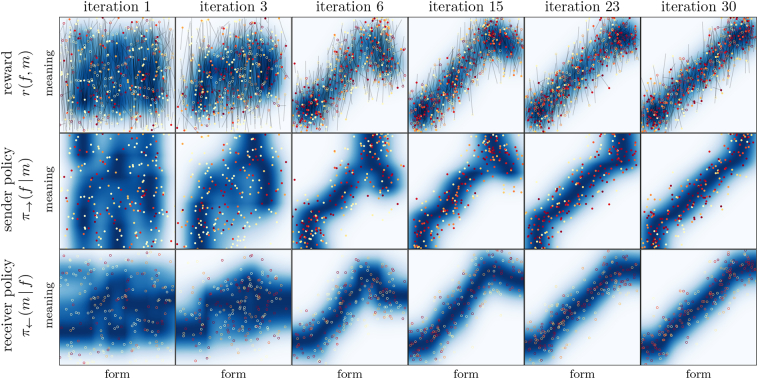
Learning progress in the 1D-1D-setting. Filled circles indicate the form-meaning pairs when the agent was sender (i.e. the form it chose to communicate the respective meaning); open circles indicate the form-meaning pairs when it was receiver (i.e. the meanings it chose as interpretation of the respective form). Grey lines indicate the corresponding form-meaning pairs of the other agent for the respective communication act. The colour of the circles indicates the reward both agents received for that specific act of communication (red: high reward; light yellow: low reward), which scales with the distance between the intended and interpreted meaning (length of the grey lines in meaning direction). A total of 400 out of 10,000 communication acts per iteration is shown. The heat maps show the expected reward (top row); the sender policy (middle row; normalisation along the form dimension; only sending form-meaning pairs shown); and the receiver policy (bottom row; normalisation along the meaning dimension; only receiving form-meaning pairs shown). The detailed learning progress for both agents is shown in the supplementary material at https://robert-lieck.github.io/emergence-of-symbols. (For interpretation of the references to colour in this figure legend, the reader is referred to the web version of this article.)

We start with a simple 1D-1D-scenario, as it was illustrated in Fig. 2. Simulations were carried out with transmission noise from a single Gaussian with a standard deviation of *σ* = 0.05. A typical learning progress is shown in Fig. 8, including the expected reward *r*(*f*, *m*), the sender policy *π*_→_(*f*| *m*), and the receiver policy *π*_←_(*m*| *f*) of one of the two agents.

The optimal policy for this scenario is a continuous one-to-one mapping between forms and meanings, which corresponds to the diagonal in the last column (iteration 30) of Fig. 8 (a diagonal in the other direction would be equally optimal).

We can observe two important characteristics of the learned signalling conventions. First, we see that over the first 6 iterations, the receiver policy becomes *unambiguous* (Zuidema and Westermann (2003) call this property *distinctiveness*). That means, for any form *f*, *π*_←_(*m*| *f*) is a unimodal low-variance distribution of meanings. An unambiguous receiver policy is crucial for a successful communication because it allows to reliably assign meaning to the received forms. If the receiver policy is not unambiguous, we speak of a *malign ambiguity* because it leads to severe misunderstandings. Intuitively, this corresponds to the same utterance having multiple substantially different meanings that cannot be disambiguated (*homonymy*).

The second important observation is that the sender policy also becomes unambiguous (high *specificity* in terms of Zuidema and Westermann (2003)), but it converges much slower than the receiver policy. In iteration 6, we still see a bimodal distribution of forms for certain meanings, that is, these meanings are communicated using very different forms. As opposed to ambiguity in the receiver policy, this does not lead to severe misunderstandings (which is also the reason why convergence is slower): If the receiver policy is unambiguous, these different forms are still mapped to the same meaning (*synonymy*). We therefore speak of a *benign ambiguity* if it occurs in the sender policy.

The reason why an unambiguous sender policy is still better than an ambiguous one is best understood from an information-theoretic perspective, as discussed in Section 7. Intuitively, the reason is that several forms are “spent” on the same meaning, which effectively reduces the number of forms that are available for communicating and differentiating other meanings. Communication is therefore not impaired by single severe misunderstandings but by many small ones.

### 6.2 Local optima

**Fig. 9:**
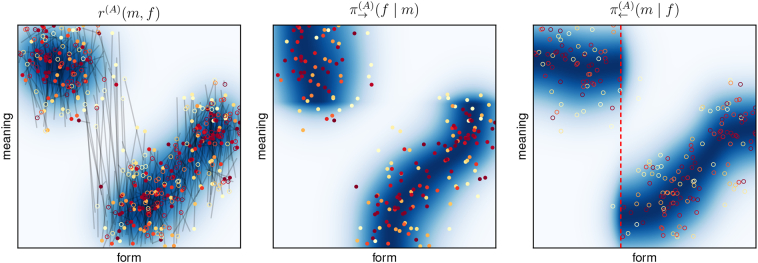
Sub-optimal signalling conventions corresponding to a local optimum in the 1D-1D-setup: (left): expected reward, (centre): sender policy, (right): receiver policy. Circles and lines as in Fig. 8.

In the example from Fig. 8, the policies eventually converged to the optimal solution, but this is not always the case. Sometimes, learning converges to a local optimum with a fragmented form space. Such an example is shown in Fig. 9 and the associated permissibility analysis is shown in Fig. 7 (it was used in Section 5.2.1 to explain the procedure). This case also corresponds to the one illustrated in Fig. 2(b).

This configuration is sub-optimal due to the discontinuous receiver policy: Around the red dashed line in the right panel of Fig. 9, the interpretation of forms suddenly changes drastically in meaning. Forms in this region are therefore subject to a malign ambiguity and cannot be used in near-optimal communication, as we can see in the permissibility analysis in Fig. 7 (again indicated by the red dashed line). Due to the exploratory policies of the agents, forms in this gap region are still chosen from time to time. This is reflected in a non-zero value of *π*_→_(*f*) at this point (grey dashed line in the lower plot of Fig. 7). The resulting misunderstandings appear as long grey lines in the left panel of Fig. 9.

#### 6.2.1 Statistical evaluation

To better understand how these sub-optimal solutions come about, whether they represent single outliers or occur frequently, and if convergence to the optimal solution can be guaranteed under certain conditions, we performed a comprehensive statistical analysis. Our focus was on testing the influence of transmission noise, as it represents the primary reason why discretisation in the receiver policy and the associated malign ambiguity are problematic (also cf. the results by Zuidema and Westermann (2003)). Our hypothesis therefore was that an increased level of noise should lead to a more robust convergence, because the effects of fragmentation become apparent early on in the learning process and can therefore be eliminated, before the agents run into a local optimum.

We tested four different conditions for the transmission noise and recorded 150 learning trajectories with 50 iterations for each of these conditions: In the **wide** condition, we used Gaussian noise with a standard deviation of *σ* = 0.2; in the **narrow** condition, the standard deviation was *σ* = 0.05; in the **mixed** condition, we used a mixture of two Gaussians with equal mixture weights (*α*_1_ = *α*_2_ = 0.5) and standard deviations *σ*_1_ = 0.05 and *σ*_2_ = 0.2; and in the **curriculum** condition, we progressively changed the mixture weights starting with the wide condition, via the mixed condition, through to the narrow condition.

The progressive change of the noise level from the wide to the narrow condition can be seen as a case of curriculum learning (Bengio, Louradour, Collobert, & Weston, 2009) and is similar to simulated annealing approaches for optimisation (Dekkers & Aarts, 1991; Kirkpatrick, Gelatt, & Vecchi, 1983). The general idea is to ensure that in the early learning phase, the agents robustly converge to the approximately correct region of the search space (the basin of attraction of one of the two globally optimal solutions), while then progressively allowing them to optimise their behaviour and converge to the exact optimum.

**Fig. 10:**
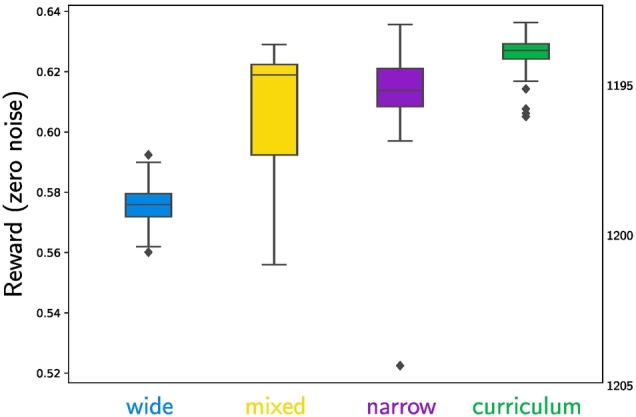
Boxplots of the average reward for the four different conditions with zero transmission noise. The curriculum condition significantly outperforms the other conditions in terms of average reward and reliability.

To be able to compare the resulting policies independently of the noise conditions under which they were learned, we performed an evaluation under zero-noise conditions at the end of each learning trajectory. The statistics of this evaluation are shown in Fig. 10 and confirm our hypothesis that the curriculum learning strategy yields the best results.

However, the *wide* condition performs worst in this evaluation. This can be expected, because the learned policies are proportional to the expected reward, which is spread out due to the wide noise during learning. Therefore, the reward under zero-noise conditions does not reveal the actual shape of the learned policies, including whether they are fragmented or not.^11^ Moreover, it does not yield any insights about the learning *process* itself.

#### 6.2.2 Embedding

**Fig. 11:**
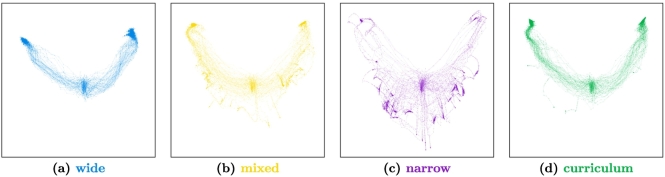
Embeddings of the learning trajectories for the four different noise conditions. See Fig. B.20 in Appendix C for more detailed plots.

To better understand characteristics of the learning process, we visualised the trajectories in a two-dimensional embedding using the *Isomap* algorithm (Tenenbaum, De Silva, & Langford, 2000). Such an embedding projects the learned policies from the high-dimensional space of all possible policies down to a point in two dimensions, while (approximately) preserving relative distances between different policies. This allows for visualising the learning trajectories as paths in a two-dimensional plot. The results for the different conditions are shown in Fig. 11. In these maps, the random initialisation is located in the centre and the two optima (the two possible diagonal mappings) are located in the top-left and top-right corner.^12^

The plots clearly show that the *wide* condition displays the most robust convergence properties towards the global optima, but also that the optima are not effectively reached. In contrast, the *narrow* condition displays the most volatile behaviour with trajectories converging to the global optima but also to many different local optima (accumulation points in the lower half of the plot). The *mixed* condition can mitigate unstable convergence only to some degree and is still regularly converging to local optima. Finally, the *curriculum* condition combines the robustness from the wide initial noise with reliable convergence to the global optima from the narrow final noise condition (except for four outliers that can also be seen in Fig. 10).

We can conclude that convergence to local optima is a potential reason for sub-optimal and fragmented signalling conventions. Furthermore, we see that the learning progress strongly depends on the external conditions, in particular, on the noise level. In this light, it is plausible to assume that one of the purposes of infant-directed speech (see e.g. Vallabha et al., 2007) is to ensure a fast and robust convergence of the children's signalling conventions to that of their particular culture.

### 6.3 Modal worlds

In our third experiment, we investigate the scenario of a modal world, as studied by Feldman (2012). That is, meanings are not uniformly distributed as in our previous examples, but instead their distribution exhibits distinct modes, where meanings are most likely to occur. This situation may, for instance, partly explain the emergence of colour words, which are not uniformly spread in the colour space, exhibit a number of cross-cultural characteristics, and correlate with the actual need for communicating about specific objects (Berlin & Kay, 1969; Gibson et al., 2017; Kay et al., 2003).

We extend this scenario by considering modes that have non-zero dimensionality, that is, they represent manifolds that are embedded into meaning space – a possibility that was already mentioned by Feldman (2012). Specifically, we consider manifolds in meaning space that locally have the same topology as the form space. Our results demonstrate that not only are the distinct modes represented by distinct symbols, but that additionally each symbol establishes a locally continuous iconic mapping to the respective mode.

Furthermore, we investigate the effect of assigning different weights to the different modes. For a discrete representation, the probability of each symbol should correspond to the probability of the respective mode. We show that this also holds for a continuous form space with embedded symbols and that, moreover, the resulting form distribution can be understood in terms of Shannon's source coding theorem (Shannon, 1948).

**Fig. 12:**
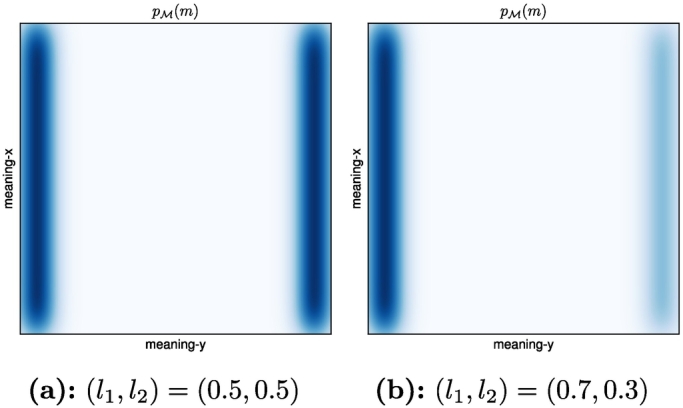
Meaning distribution *p*_ℳ_(*m*) for the two versions of the *TwoLines* world model.

**Fig. 13:**
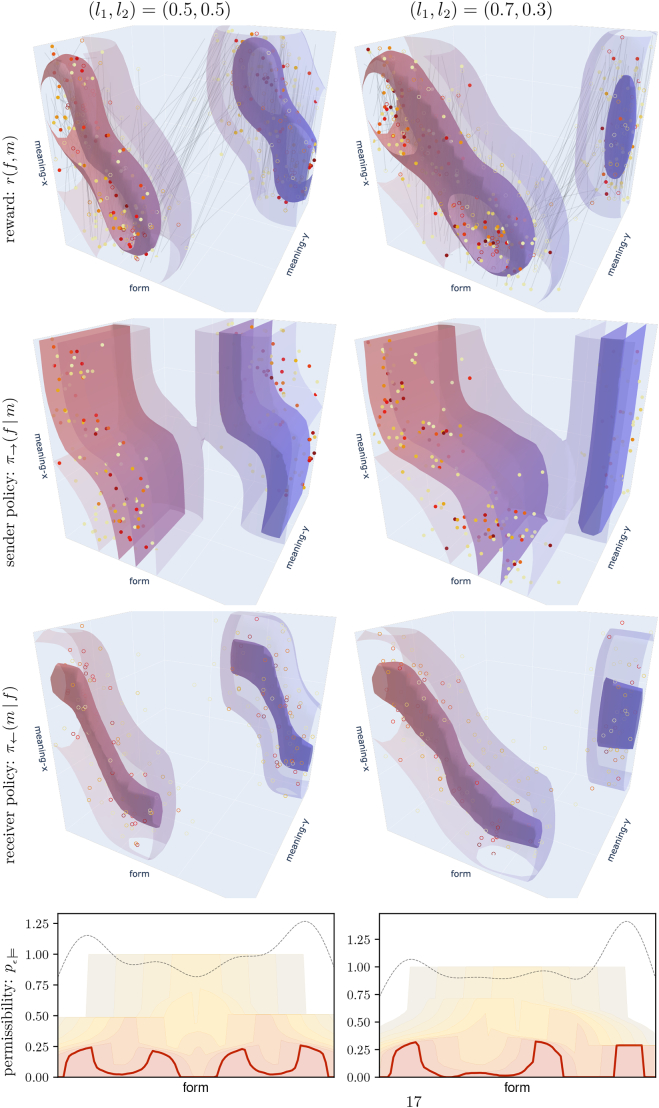
Results for two different scenarios of the *TwoLines* world model with (*l*_1_, *l*_2_) = (0.5,0.5) (left) and (*l*_1_, *l*_2_) = (0.7,0.3) (right). The **top three rows** show the expected reward, sender policy, and receiver policy, as in Fig. 8. Instead of the heat maps, we show isosurfaces at *r*(*f*, *m*) = {0.5,0.05} for the expected reward and probability levels *π*_→_(*f*| *m*) = *π*_←_(*m*| *f*) = {0.8,0.2} for the sender and receiver policies; the greater value is shown with high opacity, the smaller with low opacity in each case. The **bottom row** shows the corresponding permissibility analyses, see Section 5.2.1 for explanation. Interactive versions are available in the supplementary material at https://robert-lieck.github.io/emergence-of-symbols.

Our *TwoLines* world model consists of two one-dimensional sub-spaces embedded into a two-dimensional meaning space, while the form space is one-dimensional, as before. This setting was illustrated in Fig. 1. We sample a meaning by 1) choosing one of the two lines with probability *l*_1_ and *l*_2_, respectively, 2) sampling a meaning uniformly from that line, and 3) adding Gaussian noise with standard deviation *σ* = 0.05 to the *x*- and *y-*component. The meaning distributions for the two different scenarios with (*l*_1_, *l*_2_) = (0.5,0.5) and (*l*_1_, *l*_2_) = (0.7,0.3) are shown in Fig. 12, while Fig. 13 shows the corresponding simulation results.

For both scenarios, the permissibility analysis in the bottom row of Fig. 13 indicates two distinct symbols. The sender and receiver policies in the second and third row of Fig. 13 indicate that these symbols are associated to the two modes in meaning space, that is, the two lines of our world model. Moreover, we see that the mapping between form space and meaning space is continuous within each of the two symbols. This means that we have two distinct symbols with an internal space that represents an iconic mapping to the meaning space – a prototypical example of how we characterised discrete symbols in continuous spaces in Section 5.

Comparing the volume of the symbols in form space, we see that the symbols in the in the 0.5/0.5-scenario (on the left) have equal volume, while those in the 0.7/0.3-scenario (on the right) have a substantially different volume. The relative volume of the smaller symbol on the right is slightly less than 0.3, but, on the other hand, the probability *π*_→_(*f*) of forms to be used in this area is elevated (grey dashed line). As the probability of a symbol to be used in communication equals the product of its volume and the value of *π*_→_(*f*) in that area, this means that in both scenarios the probability of the different symbols corresponds approximately to the probability of the respective modes, as expected in analogy to the discrete case.

Considering the distribution of forms, in the 0.5/0.5-scenario this implies that forms are approximately uniformly distributed. More interestingly, due to the different volume of the symbols this is also the case in the 0.3/0.7-scenario.^13^ Such an approximately uniform (maximum entropy) distribution in form space corresponds to what one would expect based on the source coding theorem (MacKay, 2003; Shannon, 1948). This suggests the agents learn an optimal source coding adapted to the respective world, which is in line with existing theoretical models and empirical studies on information density in language (Jaeger & Levy, 2007; Piantadosi, Tily, & Gibson, 2011; Zaslavsky, Kemp, Regier, & Tishby, 2018). We will discuss the information-theoretic perspective in more detail in Section 7.

**Fig. D.21:**
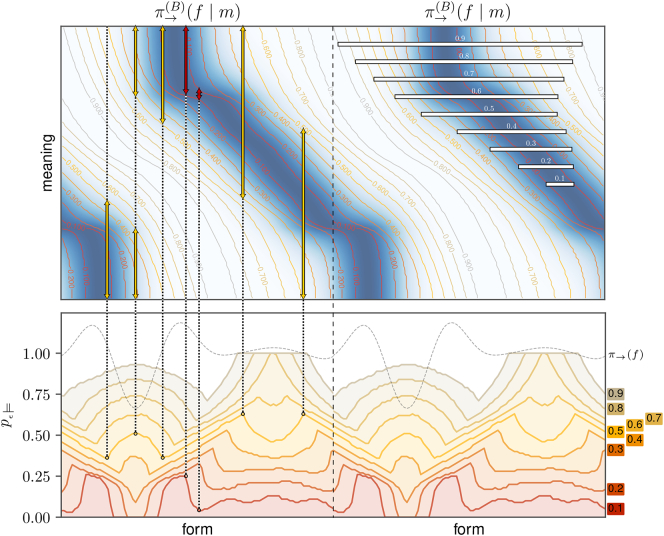
Characteristic points of *p*_*ε*__⊨_(*f*) in permissibility analysis.

Finally, we make two observations for which we do not have an obvious explanation. First, in near-optimal policies with *ε* = 0.1, forms towards the boundary of symbols are much more likely to occur (also see Fig. D.21 in Appendix E). This might be related to fewer data at the boundaries of meaning space due to the way we ensure boundedness of forms and meanings via rejection (see end of Section 4.1). Together with the *L*_2_-regularisation, this results in “rounded” ends of the expected reward, which are then extrapolated towards the boundaries by the normalisation. It might also be related to the fact that agents do not distinguish between whether they were sender or receiver when collecting data and that both policies are thus derived from the same estimate of the expected reward. Second, the smaller symbol in the 0.7/0.3-scenario is effectively reduced to an atomic symbol with zero-dimensional internal meaning space. It is therefore impossible to communicate a specific location on the corresponding line. This does not seem entirely unreasonable, as that line is less important than the other. However, we wonder whether the transition between atomic and non-atomic symbols (or between one- and two-dimensional symbols in higher dimensions) is a smooth one or whether there is a phase transition leading to a collapse of near-atomic symbols.

### 6.4 Topological mismatch

**Fig. 14:**
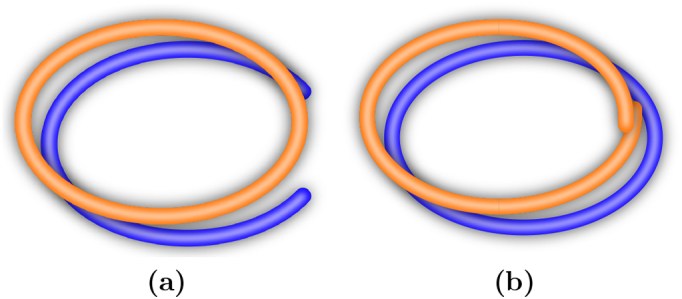
Topological mismatch between form space (orange) and meaning space (blue). (a): For a cyclic form space and linear meaning space (the case of our experiments), a malign ambiguity of the receiver policy is avoided by leaving a gap in form space, thus inducing a discretisation. (b): For a linear form space and cyclic meaning space (not shown in the experiments) the form space can be glued together to form a cycle, which only creates a benign ambiguity of the sender policy without a discretisation of the form space. (For interpretation of the references to colour in this figure legend, the reader is referred to the web version of this article.)

Our final experiment addresses the case of topological mismatch between form and meaning space. We investigated this situation in the simplest possible case, which is a 1D-1D-scenario where the form space has cyclic boundary conditions, as it was illustrated in Fig. 3. As discussed, an entirely continuous mapping is not possible in this case: The forms that happen to be mapped to the ends of the meaning space cannot be immediate neighbours in form space as this would create a malign ambiguity of the receiver policy in the presence of transmission noise. Note that the inverse case (linear form space/cyclic meaning space) is less problematic because the form space can be glued together to form a cycle, which only creates a benign ambiguity of the sender policy, as illustrated in Fig. 14.

**Fig. 15:**
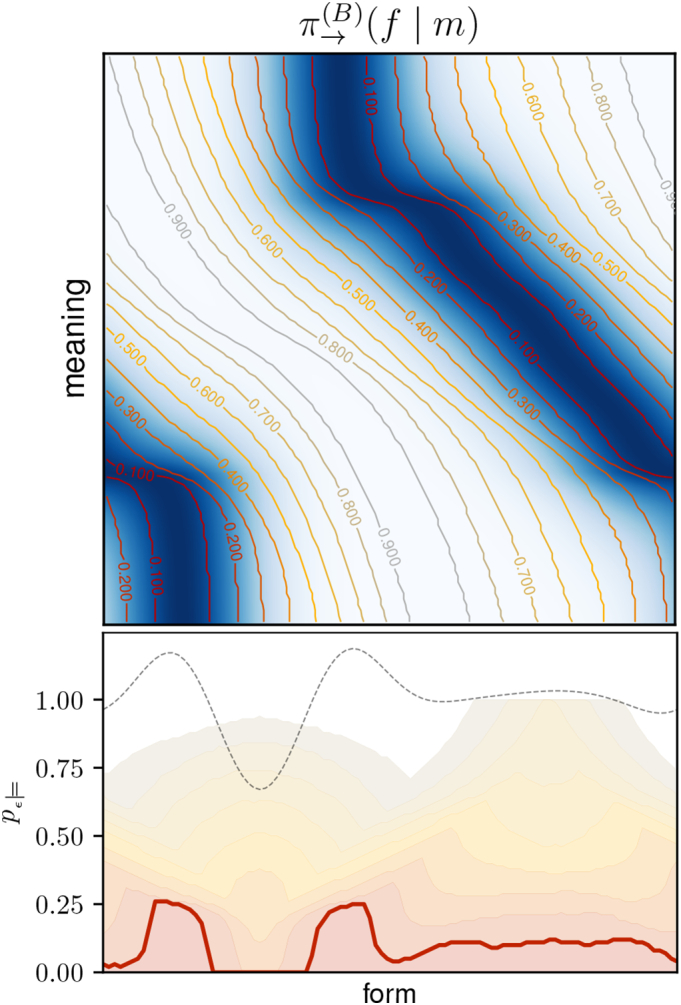
Permissibility analysis for a 1D-1D-setup with cyclic form space and a discretisation induced by the boundaries in meaning space.

**Fig. 16:**
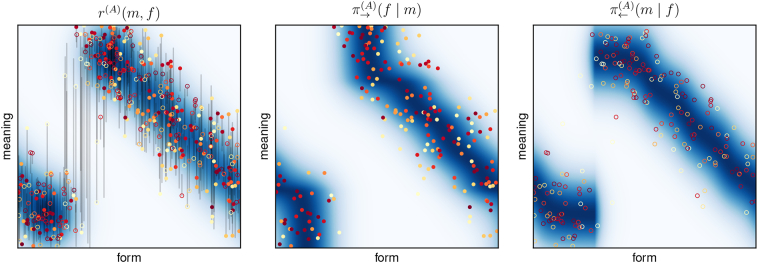
Expected reward (left), sender policy (centre), and receiver policy (right) for a 1D-1D-setup with cyclic form space. Circles and lines as in Fig. 8.

Since the form space is translational invariant due to the cyclic boundary conditions, the resulting discretisation may occur at an arbitrary location in form space. The results for this setup are shown in Fig. 16, the corresponding permissibility analysis in Fig. 15. For near-optimal communication (*ε* ≤ 0.2), a discretisation in form space is induced by the boundaries in meaning space, while the remaining form space forms one contiguous symbol with a continuous iconic mapping that is wrapped around the cyclic boundary in form space.

**Fig. 17:**
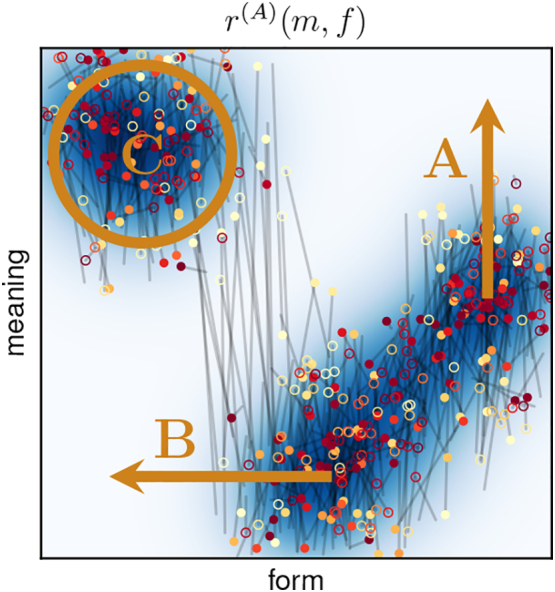
Stability of sub-optimal solutions.

These results demonstrate that a mismatch in topology between form and meaning space, indeed, leads to a discretisation in form space. This has far-reaching consequences as it explains the emergence of symbols on a fundamental and abstract level. The results are therefore of significant relevance beyond theoretical considerations and simulations and we expect real signalling systems, from language and communication in non-human animals to sign language and music, to be characteristically shaped by the interplay between the topology of the world and that of the employed form space.

In reality, both the meaning space as well as the employed form space have a highly complex topology, which can be expected to be incompatible in many ways. Furthermore, even if subsets have a matching topology, the task of finding an optimal mapping is challenging and the chances of converging to a local optimum with fragmented symbols are high. Topological mismatch is therefore a potential explanation for the emergence of discretisation in any signalling system with a continuous form space.

## 7 Information-theoretic perspective

We complement our experimental results by providing an information-theoretic interpretation of some of the observed phenomena. Our experiments can be considered an example of communication over a noisy channel (Cover & Thomas, 2006; Shannon, 1948). The agents can be understood as encoding meanings as forms, which are then transmitted and decoded by the receiving agent. However, there are two differences as compared to the conventional setup that we need to take into account.

First, the sender and receiver policy are stochastic mappings as opposed to conventional source codes, which are assumed to be deterministic. They are introducing additional noise on top of the noise from the channel for transmitting forms (*f*-*f*′-channel). We can still apply the view of source coding and data compression to better understand the sender policy because the final policies are near-deterministic mappings, but we have to separately explain how such a near-deterministic mapping comes about in the first place. To this end, it will be useful to treat the sender and receiver policy as separate noisy communication channels (*m*-*f*-channel and *f*′-*m*′-channel, respectively):

**Figure:**
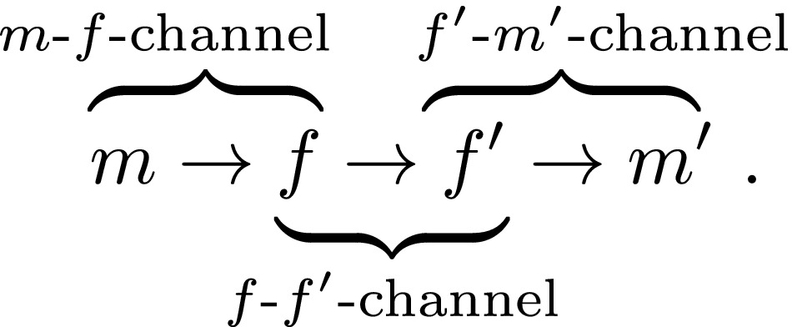

Alternatively, we can consider the compound channel of the sender or receiver policy with form transmission (*m*-*f*′-channel and *f*-*m*′-channel, respectively). Together, these channels result in a global *m*-*m*′-channel for transmitting meanings.

The second difference to conventional source coding is that the agent's objective is not to optimise data compression through the *m*-*f*-channel but to maximise communication success through the *m*-*m*′-channel as measured by the reward function. This is particularly important for understanding the impact of topology, symbol fragmentation and boundary effects.

### 7.1 Rate-distortion theory

Our setup is best understood through the lens of rate-distortion theory (Cover & Thomas, 2006; Shannon, 1959). The *rate* of a noisy communication channel is the amount of information that it can transmit, quantified as the mutual information between its input *X* and its output $\hat{X}$; the *distortion* of the channel is the expected value of a distortion measure *d* between inputs and outputs

(8) $$\text{rate:}\;I(X,\hat{X})\;\text{distortion:}\;E\left[d(X,\hat{X})\right].$$

The rate and distortion depend on the distribution *p*(*x*) over channel inputs and the channel's transmission distribution $p\left(\hat{x}|x\right)$.

Rate-distortion theory is then concerned with the question of how rate and distortion are related. That is, how much information needs to be transmitted in order to reconstruct the original signal (i.e. the forms in our case) with a limited distortion. Or, conversely and the more appropriate question in our case, what is the lowest possible distortion for a given amount of transmitted information.^14^ An advantage of rate-distortion theory is that it generalises to the continuous setting. From this perspective, our agents can be understood as trying to achieve the lowest possible distortion of the global *m*-*m*′-channel by adapting their sender and receiver policies. To this end, they also generally need to maximise the transmission rate of the individual transmission channels. We will now discuss this view in more detail.

### 7.2 Maximal transmission rate

The mutual information can be written as

(9) $$I\left(X,\hat{X}\right)=H\left(\hat{X}\right)-H\left(\hat{X}|X\right),$$

where $H\left(\hat{X}\right)$ is the entropy of the channel output and $H\left(\hat{X}|X\right)$ is the conditional entropy of the channel's transmission distribution (see Appendix D.1 for details). From this, it is clear that the transmission rate is maximal if the entropy $H\left(\hat{X}\right)$ of the channel output is maximal and the conditional entropy $H\left(\hat{X}|X\right)$ of the transmission distribution is minimal.

In an idealised case, the output distribution $p\left(\hat{x}\right)$ should therefore be uniform and the channel's transmission distribution $p\left(\hat{x}|x\right)$ should deterministically map inputs *x* to outputs $\hat{x}$. Intuitively, each of these two conditions makes sense by itself: A uniform output distribution makes optimal use of all available outputs and avoids “clumping together” inputs by mapping them to (or near to) the same output, while a deterministic mapping ensures perfect transmission without any noise. Maximising the transmission rate corresponds to the idea of an *unambiguous* sender policy, as described in Section 6.1.

#### 7.2.1 Ambiguous sender policy (benign ambiguity)

When we look at the encoding *m*-*f*-channel in the light of rate maximisation, the development of the sender policy *π*_→_(*f* |  *m*) in the 1D-1D-setup (shown in Fig. 8) seems quite natural: The conditional entropy is drastically reduced (meanings are almost deterministically mapped to forms), while the full form space is uniformly filled (ignoring boundary effects). The remaining ambiguity at an intermediate stage is eliminated more slowly because it only induces a comparatively small increase in conditional entropy (reduction in transmission rate) and does not significantly increase distortion, which is why we call this a *benign* ambiguity. In linguistic terms, this corresponds to *synonymy*, that is, multiple forms having the same meaning.

Likewise, the partitioning of the form space in the *TwoLines* world (shown at the bottom of Fig. 13) seems natural: For the 0.5/0.5-case the two symbols occupy equal volumes in form space, while for the 0.7/0.3-case the ratio is approximately 0.7/0.3. This means that in both cases forms are roughly uniformly distributed when ignoring boundary effects and the gap between the symbols (the maxima of the 0.1-, 0.2-, and 0.3-permissibility lines have equal height).

#### 7.2.2 Boundary effects

The transmission distribution *p*_ℱ_(*f*′| *f*) of forms through the *f*-*f*′-channel influences the overall transmission rate through the *m*-*m*′-channel. In our experiments, *p*_ℱ_ is relatively neutral in that it simply adds Gaussian noise to the input *f* so that all input forms are spread out equally. An exception are the boundaries of the form space (see Section 4.1): An input form that lies at the boundary can only be mapped to the same location or further inwards; it is therefore spread out less than forms at other locations. As a consequence, the conditional entropy close to the boundaries is lower and the *f*-*f*′-channel can be conceived as locally having a higher transmission rate.

To understand other phenomena, such as the separation of symbols or why a malign ambiguity in the receiver policy is eliminated more quickly than a benign ambiguity in the sender policy, we have to consider the distortion of the global *m*-*m*′-channel.

### 7.3 Minimal distortion

Distortion in our setup is measured by the reward function *ρ*. With a distortion measure of

(10) $$d\left(m,m^{\prime }\right)=1-\rho \left(m,m^{\prime }\right),$$

the agents' objective of maximising the expected reward is in fact equivalent to minimising the expected distortion of the overall *m*-*m*′-channel

(11) $$E\left[d\left(m,m^{\prime }\right)\right]=1-E\left[\rho \left(m,m^{\prime }\right)\right].$$

The transmission distribution of this channel is

(12) $$p\left(m^{\prime }|m\right)=\iint \pi_{\leftarrow }\left(m^{\prime }|f^{\prime }\right)\;p_{ℱ}\left(f^{\prime }|f\right)\;\pi_{\to }\left(f|m\right)\;\mathrm{dfd}f^{\prime },$$

where the forms *f* and *f*′, used to transmit the meaning, are marginalised out. The agents optimise this distribution (via reinforcement learning) to minimise distortion by adapting their sender and receiver policies. As a by-product, the transmission rate of the sender and receiver policy is maximised (meanings are compressed into forms) as long as this does not conflict with retaining a small distortion in one of the ways discussed below.

One source of increased distortion is of course noise, in particular, noise in the sender and receiver policy, which initially is high but is strongly reduced during the learning process, and noise during form transmission. Beyond noise, the most relevant cause for high distortion in our setup are malign ambiguities of the receiver policy, which may occur during the learning process and are the reason for the separation of symbols in case of a discontinuous receiver policy.

#### 7.3.1 Ambiguous receiver policy (malign ambiguity)

Receiver policies are preferred to be *unambiguous*, as described in Section 6.1. This already seems natural from the rate-maximisation perspective, since ambiguity leads to an increase in conditional entropy and thus to a reduction in transmission rate, as discussed above for the sender policy. However, ambiguity in the receiver policy is much more detrimental to the overall distortion than ambiguity in the sender policy. Ambiguities in the sender policy – i.e. several different forms being used to communicate the same meaning (synonymy) – can still be unambiguously decoded. In contrast, ambiguity in the receiver policy – i.e. the same form being used to communicate several different meanings (homonymy) – leads to misunderstandings, that is, a high distortion value of the *m*-*m*′-channel. This is the technical reason why the temporary benign ambiguity in the sender policy in our example from Fig. 8 can persist over a significant period of time during learning, while a malign ambiguity in the receiver policy would vanish quickly.

#### 7.3.2 Discontinuous receiver policy

The second situation leading to high distortion are discontinuities in the receiver policy. This means that slight changes in form space may result in drastic changes of the interpreted meaning. These discontinuities are problematic due to the continuous topology of the form and meaning spaces that is inherent in our entire setup. The transmission noise *p*_ℱ_ of the *f*-*f*′-channel is bound to the topology of the form space: Forms that are close are more likely to be “mixed up” during transmission. The reward function *ρ* (and thus the distortion measure *d*) are bound to the topology of the meaning space: Meanings that are far result in a low reward and a high distortion value. Moreover, any additional noise induced by the sender and receiver policy also reflects proximity in form and meaning space.

In the presence of transmission noise, discontinuities of the receiver policy result in an *induced* malign ambiguity: If a form lies close to a discontinuity, it has high chances of ending up “on the other side” due to transmission noise and consequently being interpreted to have a substantially different meaning. This situation is comparable to inherent malign ambiguities of the receiver policy – after all, to the distortion measure it does not matter whether transmission is faithfully and interpretation ambiguous or the interpretation is faithful and transmission ambiguous.

This situation is solved by “leaving a margin” in form space, that is, by a separation of symbols: On both sides of the discontinuity there has to be a region in form space that is still mapped to the “old” meaning but that is not actively used by the sender policy. In this way, forms that happen to end up in the margin are still correctly interpreted. The size of this margin is essentially determined by the amount of transmission noise and has to be balanced against the caused reduction in transmission rate due to the effectively smaller form space.

### 7.4 Stability of sub-optimal solutions

The stability of sub-optimal solutions, that is, the fact that an unnecessarily fragmented form space may represent a locally optimal solution, can also be understood from a rate-distortion perspective. Take the example from Fig. 7, Fig. 9. In this case, a continuous one-to-one mapping (as in Fig. 8) would be the optimal solution, but one end of the meaning space ended up being mapped to the “wrong” end of the form space and vice versa. To understand why this is a locally optimal solution, we can proceed by formulating a “proof by contradiction”. That is, we assume that it was in fact *not* a local optimum, which means that there should be some path to the global optimum, along which communication constantly improves. If, on the contrary, all possible paths to the global optimum turn out to (temporarily) deteriorate communication, the fragmented solution must be locally optimal.

In particular, to arrive at the global optimum, we have to (A) extend the mapping to cover the full meaning space, using (B) the full form space, while (C) eliminating the smaller fragment (see Fig. 17). Throughout this process, we have to either constantly eliminate a source of distortion or increase the transmission rate.

We cannot use the entire form space (B) before eliminating C because this would create a malign ambiguity in the receiver policy and thus lead an increase in distortion. We also cannot eliminate C before extending to the full meaning space (A) because temporally the meanings corresponding to C could then not be appropriately communicated any more. But we also cannot extend to the full meaning space first (A), because this would create a benign ambiguity in the sender policy and thus a (slight) decrease in transmission rate.

Finally, we could try to perform A, B, and C at the same time, which would progressively shrink C until being eliminated. The problem with this procedure is that C needs its margin in form space in order to be functional in communication. The width of this margin and of C itself is determined by the noise in form transmission. Furthermore, C occupies the boundary of the form space, which has a particularly high transmission rate due to the boundary effect. We therefore cannot shrink C beyond a certain limit without increasing distortion.

Since all possible paths to the global optimum temporally decrease the quality of communication, the fragmented solution corresponds to a local optimum, which explains why it is a stable fixed point in our simulations.

## 8 Conclusion

We investigated the emergence of discrete symbols embedded into continuous form spaces from a theoretical and experimental perspective. We provided a rigorous definition of discrete symbols in an entirely continuous setting that unifies discrete and continuous aspects and may serve as the basis for theoretical and empirical analyses.

By simulating the learning process of two agents that acquire a shared signalling system, we empirically confirmed three causes for discretisation: 1) modal worlds, as suggested by Feldman (2012), 2) convergence to local optima with a fragmented form space, and 3) a topological mismatch between form and meaning space, as conjectured by de Boer and Verhoef (2012) and Zuidema and Westermann (2003).

First, we established general characteristics of optimal signalling conventions, in particular, the avoidance of ambiguity, differentiating between *benign* ambiguity of the sender policy and *malign* ambiguity of the receiver policy (also cf. *synonymy*/*homonymy* in linguistics and *specificity*/*distinctiveness* in (Zuidema & Westermann, 2003)).

In the case of modal worlds, we additionally showed that the distinct symbols may establish a locally continuous iconic mapping to the respective mode, which is used to represent points of the manifold of that mode. Furthermore, our results reveal that the learned policies exhibit typical properties of an optimal source coding that is learned by the agents.

To better understand the reasons for convergence to local optima with a fragmented form space, we performed a comprehensive statistical analysis, demonstrating that an increased level of transmission noise leads to more robust convergence. It is shown that a curriculum learning approach that transitions from high to low noise level reliably leads to globally optimal signalling conventions.

We investigated the situation of a topological mismatch using a cyclic form space and a non-cyclic meaning space. Our results show that the boundaries in meaning space induce a discretisation in form space and demonstrate why a discretisation in form space may also be observed in case of a continuous meaning space. This topological argument explains the emergence of discretisation on a fundamental and abstract level and it can be expected that any signalling system is characteristically shaped by the topology of the corresponding form and meaning space.

Finally, we drew the connection to information theory and in particular rate-distortion theory, which allowed for a more thorough understanding of many of the observed effects.

The joint treatment of discrete and continuous properties based on a rigorous definition of discrete symbols in continuous form spaces allows us to model the emergence of discretisation as well as the coexistence, coevolution and interplay of discrete and continuous properties. These aspects are not only relevant to human language but also to other forms of communication, such as music and signalling systems of non-human animals.

Our results shed light on an assumption – the existence of discrete symbols – that underlies a large body of research concerned with the emergence of syntactic structures and meaning in language evolution. By theoretically describing and empirically reproducing the emergence of discrete symbols in regions with continuous semantics based on simulations from first principles, we hope to provide the basis for better understanding the interplay between discretisation and continuity in communication.

## Figures and Tables

**Fig. A.18:**
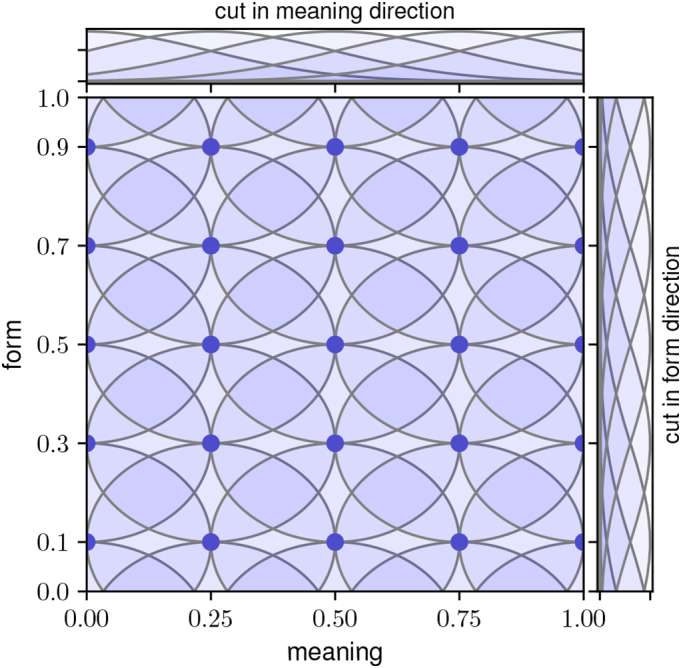
Gaussian radial basis functions for modelling the expected reward *r*(*f*, *m*) on a 5 × 5 grid. The centers are marked by blue dots; the semi-transparent ellipsoids indicate the distance of one standard deviation from the center (i.e. the inflection point). The plots on the right and the top show a one-dimensional cut along the form and the meaning dimension, respectively. Note that the form dimension is cyclic while the meaning dimension is not, leading to different grid spacings and locations of the basis function as well as cyclic overlap along the form dimension. (For interpretation of the references to colour in this figure legend, the reader is referred to the web version of this article.)

**Fig. B.19:**
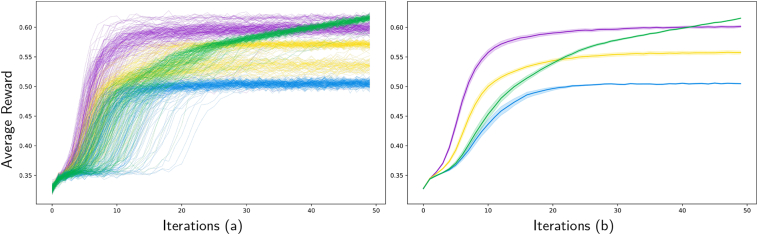
Average reward over the learning phase for the four different conditions (wide, mixed, narrow, curriculum). (a): Each thin line corresponds to one of 150 distinct learning trajectories per condition. In each iteration, the average reward over all 10,000 communication acts was taken. (b): Mean of the trajectories in (a) with error bands indicating the standard error of the mean, computed via bootstrapping.

**Fig. B.20:**
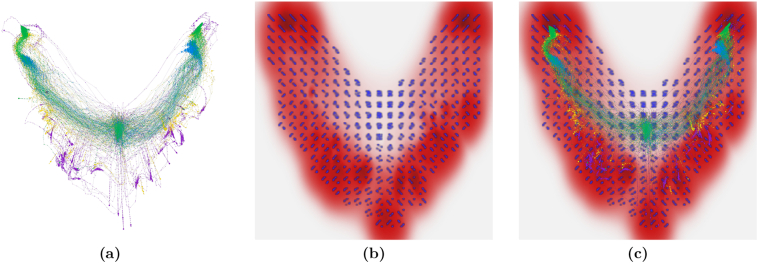
Detailed plot for the embeddings shown in Fig. 11. (a): Overlay of the four different conditions (wide, mixed, narrow, curriculum). (b): The red density plot in the background visualises the average reward at the corresponding location with dark red corresponding to the maximum average reward (the global optima in the top-right and top-left corner are clearly visible). The tiling shows the expected reward *r*(*f*, *m*) as modelled by the agent as a 11 × 11 miniature heat map plot, placed on a fixed grid. At each location the nearest neighbour (taken from all trajectories) is plotted. (c): Overlay of (a) and (b). (For interpretation of the references to colour in this figure legend, the reader is referred to the web version of this article.)

## Appendix A Expected reward

The expected reward *r*(*f*, *m*) is modelled as a linear combination of Gaussians

(A.1) $$r\left(f,m\right)=\sum_{i}\beta_{i}\;N\left(x;\mu_{i},\Sigma \right),$$

where $N\left(x;\mu ,\Sigma \right)$ is the multivariate normal distribution with mean *μ* and covariance matrix Σ, *β*_*i*_ > 0 is the weight of the *i*^th^ basis function centered at *μ*_*i*_, and

(A.2) x=f⊕m∈ℱ×ℳ

is the (*d*_*f*_ + *d*_*m*_)-dimensional vector obtained by concatenating the form *f* and the meaning *m*.

The basis functions are placed on an equidistant grid and have a diagonal covariance matrix with standard deviations equal to the grid spacing along the respective dimension, as illustrated in Fig. A.18. The large overlap between adjacent basis functions ensures a smooth shape of the expected reward *r*(*f*, *m*).

Due to the diagonal covariance matrices with fixed standard deviations for each dimension, *r*(*f*, *m*) can be computed as

(A.3) $$r\left(f,,,m\right)=\sum_{i_{1}=1}^{N_{1}}\cdots \sum_{i_{D}=1}^{N_{D}}\beta_{\left(i_{1},\ldots ,i_{D}\right)}\prod_{d=1}^{D}N\left(x_{d};\mu_{\left(i_{1},\ldots ,i_{D}\right)},,,\sigma_{d}^{2}\right),$$

where *D* = *d*_*m*_ + *d*_*f*_ is the dimension of the joint form-meaning space ℳ × ℱ, *μ*_(*i*1,…,*iD*)_ specifies the location of the basis function at grid index (*i*_1_, …, *i*_*D*_), *β*_(*i*1,…,*iD*)_ is the corresponding weight, and *σ*_*d*_ is the standard deviation for dimension *d*.

Sampling from the sender and receiver policies, *π*_→_(*f*| *m*) and *π*_←_(*m*| *f*), can be done by first sampling a mixture component with an appropriate probability and then sampling from that mixture component. Due to the diagonal covariance matrices, the probability for sampling a mixture component, which has to take conditioning on *f* or *m* into account, can be computed analytically by solving the integrals in (2) and (3) separately for each affected dimension.

## Appendix B Monte-Carlo approximation of *p*_*ε*__⊨_

We are interested in the probability *p*_*ε*__⊨_(*f*) of a form to be *ε*-permissible in communication, which requires marginalising over possible meanings

(B.1) $$p_{\varepsilon ⊨}\left(f\right)=\int_{M}〚f_{\varepsilon }⊨m〛\;p_{M}\left(m\right)\;\mathrm{dm},$$

where *p*_ℳ_ is the distribution of meanings. This integral can not generally be solved in closed form. Furthermore, evaluating *f*_*ε*_⊨*m* involves another integral (see (4)) over the form space, which again is not generally tractable. We therefore approximate both integrals with finite sums

(B.2) $$p_{\varepsilon ⊨}\left(f\right)\approx \frac{1}{N_{M}}\sum_{i=1}^{N_{M}}〚f_{\varepsilon }⊨m〛\;\text{with}\;m_{i}\sim p_{M}$$

(B.3) $$=\frac{1}{N_{ℳ}}\sum_{i=1}^{N_{ℳ}}〚\frac{1}{|ℱ|}\int_{ℱ}〚r\left(f^{\prime },m_{i}\right)\geq r(f,m_{i})〛df^{\prime }\leq \varepsilon 〛$$

(B.4) $$\approx \frac{1}{N_{ℳ}}\sum_{i=1}^{N_{ℳ}}〚\frac{1}{N_{ℱ}}\sum_{j=1}^{N_{ℱ}}〚r\left(f_{j},m_{i}\right)\geq r(f,m_{i})〛\leq \varepsilon 〛,$$

where in (B.2) we replaced the expectation over meanings with a Monte-Carlo sample of *N*_ℳ_ meanings drawn from *p*_ℳ_, and in (B.4) we replaced the integral over forms with a uniformly distributed set of *N*_ℱ_ forms. As sampling from *p*_ℳ_ is required in any case for performing the simulations, the approximation in (B.2) can always be computed, even if the integral is intractable. For practical purposes, we use a fixed set of forms on an evenly spaced grid, which increases computational efficiency and facilitates visualization.

## Appendix C Convergence

Fig. B.19 shows the development of the reward throughout the learning process for all four conditions. The final policies of each trajectory were reevaluated under zero-noise conditions to produce the boxplots in Fig. 10. We see that while all trajectories converged to a stable reward, the resulting values for the final reward are substantially different and for two out of the four conditions there are two accumulation points instead of just one, indicating the presence of local optima. The *wide* condition reliably converges to a comparatively low average reward of 0.5. In contrast, the *narrow* condition converges to a substantially better average reward of 0.6. However, closer inspection reveals that there are two accumulation points with the majority of trajectories converging to an average reward of 0.59 and a smaller number converging to an average reward of 0.61. A similar observation can be made for the *mixed* condition with accumulation points at 0.52 and 0.56. Here, the majority of trajectories converges to the higher value. Finally, the *curriculum* condition employing a transition from the wide the narrow noise distribution displays a robust convergence (with the exception of four outliers) to an average reward of 0.61, corresponding to the higher mode of the *narrow* condition. The dependency of the reward on the noise distribution is the reason for the qualitatively different shape in case of the *curriculum* learning approach as here the noise distribution changes over the course of the learning phase.

To gain better insights into why the *curriculum* condition performs significantly better and where the bimodal distributions for the *narrow* and the *mixed* condition originate from, we computed a two-dimensional embedding of the learning trajectories using the *Isomap* algorithm (Tenenbaum et al., 2000), as already shown in Fig. 11. Fig. B.20 allows for a more detailed interpretation by showing an overlay of the four different conditions, adding the reward as heat map, and miniature plots for the different locations in the embedding. For each iteration of each learning trajectory we computed the expected reward *r*(*f*, *m*) as modelled by the agent on a discrete 11 × 11 grid (some examples are displayed as blue density maps at equally spaced locations in the background in Fig. B.20(b) and (c)). The corresponding vectors of length 121 were used to compute the two-dimensional embedding.

These results reveal that the lower modes of the *narrow* and *mixed* condition correspond to local optima where the mapping from forms to meanings is fragmented into two separate symbols, similar to the situation shown in Fig. 9. The curriculum learning approach with a transition from a wide to a narrow noise distribution can therefore be understood as a means to ensure the agents to converge to the right basin of attraction (of one of the two globally optimal solutions) in the early learning phase, while progressively allowing for better convergence to the actual optimum by reducing transmission noise in the late learning phase.

## Appendix D Properties of mutual information

### D.1 Mutual information and (conditional) entropy

The transmission rate or mutual information can be written as

(D.1) $$I\left(X,\hat{X}\right)=\iint p\left(x,\hat{x}\right)\log\frac{p\left(x,\hat{x}\right)}{p\left(x\right)\;p\left(\hat{x}\right)}\;d\hat{x}\;\mathrm{dx}$$

(D.2) $$=-\iint p\left(x,\hat{x}\right)\log p\left(\hat{x}\right)\;d\hat{x}\;\mathrm{dx}$$

(D.3) $$+\iint p\left(x,\hat{x}\right)\log\frac{p\left(x,\hat{x}\right)}{p\left(x\right)}\;d\hat{x}\;\mathrm{dx}$$

(D.4) $$=-\int p\left(\hat{x}\right)\log p\left(\hat{x}\right)\;d\hat{x}$$

(D.5) $$+\int p\left(x\right)\int p\left(\hat{x}|x\right)\log p\left(\hat{x}|x\right)\;d\hat{x}\;\mathrm{dx}$$

(D.6) $$=H\left(\hat{X}\right)-H\left(\hat{X}|X\right),$$

where $H\left(\hat{X}\right)$ is the entropy of the channel output and $H\left(\hat{X}|X\right)$ is the conditional entropy of the channel's transmission distribution. The transmission rate is maximal if the entropy $H\left(\hat{X}\right)$ of the channel output is maximal and the conditional entropy $H\left(\hat{X}|X\right)$ of the transmission distribution is minimal. The conditional entropy is minimal if for each channel input (weighted by its probability of occurrence *p*(*x*)) the entropy of the channel output $p\left(\hat{x}|x\right)$ is minimal, that is, the mapping is near-deterministic.

### D.2 Invariance of the mutual information

We have made the argument that our experiments reveal general properties of communicating agents, even though our form and meaning spaces are restricted to unit cubes, because any form or meaning space of interest can be remapped to the unit cube (of sufficiently high dimensionality) by a suitable change of variables. To see that our argument is consistent, it is important to note that the mutual information (and thus the transmission rate of a channel) is invariant under such a change of variables.

Let *x* and *y* be random variables, jointly distributed as *p*(*x*, *y*). Then the transformed variables

(D.7) $$x^{'}=g\left(x\right),y^{'}=h\left(y\right)$$

with inverse transforms

(D.8) $$x=g^{-1}\left(x^{'}\right)=\bar{g}\left(x^{'}\right),y=h^{-1}\left(y^{'}\right)=\bar{h}\left(y^{'}\right)$$

are distributed as

(D.9) $$p^{\prime }\left(x^{\prime },y^{\prime }\right)=p\left(\bar{g}\left(x^{\prime }\right),\bar{h}\left(y^{\prime }\right)\right)\left|\frac{\partial \bar{g}\left(x^{\prime }\right)}{\partial x^{\prime }}\right|\left|\frac{\partial \bar{h}\left(y^{\prime }\right)}{\partial y^{\prime }}\right|$$

(D.10) $$=p\left(x,y\right)J_{x}\left(x^{\prime }\right)J_{y}\left(y^{\prime }\right)$$

(D.11) $$p^{\prime }\left(x^{\prime }\right)=p\left(x\right)J_{x}\left(x^{\prime }\right)$$

(D.12) $$p^{\prime }\left(y^{\prime }\right)=p\left(y\right)J_{y}\left(y^{\prime }\right),$$

where the Jacobians of the inverse transform

(D.13) $$J_{x}\left(x^{\prime }\right)=\left|\frac{\partial \bar{g}\left(x^{\prime }\right)}{\partial x^{\prime }}\right|=\left|\frac{\partial x}{\partial x^{\prime }}\right|$$

(D.14) $$J_{y}\left(y^{\prime }\right)=\left|\frac{\partial \bar{h}\left(y^{\prime }\right)}{\partial y^{\prime }}\right|=\left|\frac{\partial y}{\partial y^{\prime }}\right|$$

ensure preservation of probability mass under the transformation – the Jacobians essentially describe how the volume is stretched and distorted by the transformation. The Jacobians of the forward transform are related as

(D.15) $$J_{x^{\prime }}\left(x\right)=\left|\frac{\partial g\left(x\right)}{\partial x}\right|=\left|\frac{\partial x^{\prime }}{\partial x}\right|=J_{x}^{-1}\left(x^{\prime }\right)$$

(D.16) $$J_{y^{\prime }}\left(y\right)=\left|\frac{\partial h\left(y\right)}{\partial y}\right|=\left|\frac{\partial y^{\prime }}{\partial y}\right|=J_{y}^{-1}\left(y^{\prime }\right).$$

When performing such a change of variables in an integral of an arbitrary function *f* we have

(D.17) $$\iint \mathrm{dx}\;\mathrm{dyf}\left(x,y\right)$$

(D.18) $$=\iint dx^{\prime }dy^{\prime }f\left(\bar{g}\left(x^{\prime }\right),\bar{h}\left(y^{\prime }\right)\right)J_{x}\left(x^{\prime }\right)J_{y}\left(y^{\prime }\right)$$

(D.19) $$=\iint dx^{\prime }dy^{\prime }f^{\prime }\left(x^{\prime },y^{\prime }\right)J_{x}\left(x^{\prime }\right)J_{y}\left(y^{\prime }\right)$$

and conversely

(D.20) $$\iint dx^{\prime }dy^{\prime }f^{\prime }\left(x^{\prime },y^{\prime }\right)$$

(D.21) $$=\iint \mathrm{dx}\;\mathrm{dy}\;f^{\prime }\left(g\left(x\right),h\left(y\right)\right)J_{x^{\prime }}\left(x\right)J_{y^{\prime }}\left(y\right)$$

(D.22) $$=\iint \mathrm{dx}\;\mathrm{dy}\;f\left(x,y\right)J_{x}^{-1}\left(x^{\prime }\right)J_{y}^{-1}\left(y^{\prime }\right),$$

where again the Jacobians account for the distortion of the volume that is being integrated over. For the mutual information of *x*′ and *y*′ we then get

(D.23) $$I\left(x^{\prime },y^{\prime }\right)=\iint dx^{\prime }dy^{\prime }p^{\prime }\left(x^{\prime },y^{\prime }\right)\log\frac{p^{\prime }\left(x^{\prime },y^{\prime }\right)}{p^{\prime }\left(x^{\prime }\right)p^{\prime }\left(y^{\prime }\right)}$$

(D.24) $$=\iint \mathrm{dx}\;\mathrm{dy}\;p\left(x,y\right)\log\frac{p\left(x,y\right)}{p\left(x\right)p\left(y\right)}$$

(D.25) $$=I\left(x,y\right),$$

where, upon change of variables, the Jacobians in the log and those before the log all cancel out. The mutual information is thus invariant under a transformation of the variables.

## Appendix E Discontinuities of *p*_*ε*__⊨_(*f*)

For a given form *f*, *p*_*ε*__⊨_(*f*) is a function of *ε*

(E.1) $$p_{\varepsilon ⊨}\left(f\right)=\int_{M}〚f_{\varepsilon }⊨m〛p_{M}\left(m\right)\;\mathrm{dm}$$

(E.2) $$=\int_{ℳ}〚\psi \left(m,f\right)\leq \varepsilon \;〛p_{ℳ}\left(m\right)\;\mathrm{dm}$$

with

(E.3) $$\psi \left(m,f\right)=\frac{1}{|ℱ|}\int_{ℱ}〚r\left(f^{\prime },m\right)>r(f,m)〛df^{\prime }$$

(E.4) $$=\frac{|{ℱ}_{\left(m,f\right)}^{\prime }|}{|ℱ|}$$

measuring the relative volume of all forms *f*′ ∈ ℱ′_(*m*,*f*)_ with an expected reward greater than that of *f*. *p*_*ε*__⊨_(*f*) is discontinuous for a value *ε* = *ε*^∗^ if an infinitesimal change of *ε* around *ε*^∗^ causes 〚*ψ*(*m*, *f*) ≤ *ε*〛 to change value on a finite volume in meaning space. This is the case if there exists a meaning *m*^∗^ for which the gradient of *ψ*(*m*, *f*) in *m-*direction is zero and *ψ*(*m*^∗^, *f*) has a value of *ε*^∗^

(E.5) $$\exists_{m^{*}\in ℳ}\text{such}\;\text{that}\;\nabla_{m}\;\psi \left(m^{*},f\right)=0$$

(E.6) $$\text{and}\;\psi \left(m^{*},f\right)=\varepsilon^{*}.$$

Intuitively this means that when *m* changes slightly around *m*^∗^, ℱ′_(*m*,*f*)_ may change its shape but does not change its volume. Thus, when integrating over *m*, the whole region around *m*^∗^ is added at once when changing the threshold above *ε*^∗^, which causes a discontinuity in *p*_*ε*__⊨_(*f*).

While there may be complex situations in which this condition holds, there is an intuitive special case if the form space is one-dimensional and the volume enclosed by ℱ′_(*m*,*f*)_ corresponds to a simple interval [*f*, *f*′] along the form dimension with *r*(*f*, *m*) = *r*(*f*′, *m*). In this case, (50) implies that ∣*f*′ − *s* ∣ = *ε*^∗^ ∣ ℱ∣ and (E.5) means that the isosurface of the expected reward that goes through (*f*, *m*^∗^) and (*f*′, *m*^∗^) is parallel at *m*^∗^. If the meaning space is onedimensional this means that *r*(*f*, *m*) and *r*(*f*′, *m*) have the same slope in meaning-direction. This is also illustrated in Fig. D.21.

## Appendix F Supplementary data

Supplementary material Image 1

## Footnotes

1 This is crucial for the phenomenon of self-referentiality, a topic that we leave aside in this paper.

2 The option to refer to a colour as being “brick-red” is yet another interesting alternative, which makes use of compositionality in conjunction with referentiality. But these powerful options offered by human language are not the focus of this paper.

3 Here the term *symbol* is used in the more technical sense, meaning an element from a countable and usually finite set (called the alphabet), such as a natural number or a letter.

4 We will generally speak of distributions, as in the discrete setting, but note that technically we are dealing with probability *densities* in most cases.

5 Technically, this method is known as rejection sampling (Robert & Casella, 2004).

6 Each agent has to learn two mappings (sender/receiver), which have to be pairwise consistent between agents. Starting with *n* = 3 agents, we have at least as many constraints as we have variables (*n*(*n* − 1) ≥ 2*n*), which ensures consistency within the population even without explicitly enforcing self-consistency.

7 For an overview of reinforcement learning, see (Sutton & Barto, 2018); for other, more biologically inspired learning strategies, see e.g. (Baronchelli, Felici, Loreto, Caglioti, & Steels, 2006; De Vylder & Tuyls, 2006; Oliphant & Batali, 1997).

8 Importantly, during the learning process, any policy can only be *locally* optimal at the current stage of the learning process with the current estimate of the expected reward.

9 The term *ε-optimal* is sometimes used differently than defined here, namely, to refer to a policy that takes the optimal decision with probability 1-*ε* and a uniformly random decision with probability *ε*.

10 It is important to use the expected reward itself or the *sender* policy, where normalisation runs over forms. The *receiver* policy does not work because the normalisation mixes up reward values for different meanings.

11 Note that the narrow and the curriculum condition terminate with the same noise conditions and the zero-noise rewards therefore *are* comparable.

12 In Appendix C, we provide more detailed plots that include a heat map of the rewards during learning as well as the actual policies for the different locations (Fig. B.20). We also show the development of the reward throughout the learning progress in Fig. B.19, which complements the static box plot in Fig. 10 and also reveals accumulation points corresponding to the local optima.

13 In fact, the policies shown in the permissibility analysis at the bottom of Fig. 13 are not actually uniform: neither the *ε*-optimal policies (different lines of $p_{\varepsilon ⊨}\left(f\right)$), nor the agent's policy *π*_→_(*f*). However, there are several accumulation points of $p_{\varepsilon ⊨}\left(f\right)$. The “clean” ones for high values of *ε* > 0.3 correspond to the mode's probabilities (0.5 on the left; 0.7 and 0.3 on the right). But another accumulation point for near-optimal policies (*ε* ≤ 0.3) lies around 0.25. It can be found for *both* symbols in *both* scenarios and is the reason why we speak, somewhat inaccurately, of uniformly distributed forms.

14 Importantly, the transmission rate of a channel is invariant under reparameterisation, which means that we do not loose generality by assuming unit cubes as form and meaning spaces (see Appendix D.2 for details).

## References

[bb0005] Baronchelli A., Felici M., Loreto V., Caglioti E., Steels L. (2006). Sharp transition towards shared vocabularies in multi-agent systems. Journal of Statistical Mechanics: Theory and Experiment.

[bb0010] Bengio Y., Louradour J., Collobert R., Weston J. (2009). Proceedings of the 26th annual international conference on machine learning.

[bb0015] Berlin B., Kay P. (1969).

[bb0020] Bishop C.M. (2007).

[bb0025] Bleys J., Loetzsch M., Spranger M., Steels L. (2009). The grounded colour naming game. Institute of Electrical and Electronics Engineers.

[bb0030] de Boer B. (2000). Self-organization in vowel systems. Journal of Phonetics.

[bb0035] de Boer B. (2012). The evolution of language.

[bb0040] de Boer B., Verhoef T. (2012). Language dynamics in structured form and meaning spaces. Advances in Complex Systems.

[bb0045] Brighton H., Kirby S. (2006). Understanding linguistic evolution by visualizing the emergence of topographic mappings. Artificial Life.

[bb0050] Cangelosi A., Parisi D. (2002). Simulating the evolution of language.

[bb0055] Chandler D. (2017).

[bb0060] Chew E. (2000).

[bb0065] Christiansen M.H., Kirby S. (2003).

[bb0070] Cohn R. (1997). Neo-riemannian operations, parsimonious trichords, and their " tonnetz" representations. Journal of Music Theory.

[bb0075] Cover T.M., Thomas J.A. (2006).

[bb0080] De Jong E.D. (1999). European conference on artificial life.

[bb0085] De Vylder B., Tuyls K. (2006). How to reach linguistic consensus: A proof of convergence for the naming game. Journal of Theoretical Biology.

[bb0090] Dekkers A., Aarts E. (1991). Global optimization and simulated annealing. Mathematical Programming.

[bb0095] Dimos K., Dick L., Dellwo V. (2015).

[bb0100] Eimas P.D., Siqueland E.R., Jusczyk P., Vigorito J. (1971). Speech perception in infants. Science.

[bb0105] Ellison T.M. (2013). Algorithmic probability and friends. Bayesian prediction and artificial intelligence.

[bb0110] Euler L. (1739).

[bb0115] Feldman J. (2012). Symbolic representation of probabilistic worlds. Cognition.

[bb0120] Gärdenfors P. (2004).

[bb0125] Gibson E., Futrell R., Jara-Ettinger J., Mahowald K., Bergen L., Ratnasingam S., Gibson M., Piantadosi S.T., Conway B.R. (2017). Color naming across languages reflects color use. Proceedings of the National Academy of Sciences.

[bb0130] Hastie T., Tibshirani R., Friedman J. (2008).

[bb0135] Hurford J.R. (1989). Biological evolution of the Saussurean sign as a component of the language acquisition device. Lingua.

[bb0140] Jaeger T.F., Levy R.P. (2007). Advances in neural information processing systems.

[bb0145] Janik V.M. (2009). Acoustic communication in delphinids. Advances in the Study of Behavior.

[bb0150] Janik V.M., Sayigh L.S., Wells R.S. (2006). Signature whistle shape conveys identity information to bottlenose dolphins. Proceedings of the National Academy of Sciences.

[bb0155] Janik V.M., Slater P.J. (1997). Vocal learning in mammals. Advances in the Study of Behaviour.

[bb0160] Juslin P.N., Laukka P. (2003). Communication of emotions in vocal expression and music performance: Different channels, same code?. Psychological Bulletin.

[bb0165] Kay P., Berlin B., Maffi L., Merrifield W.R. (2003).

[bb0170] Kirkpatrick S., Gelatt C.D., Vecchi M.P. (1983). Optimization by simulated annealing. Science.

[bb0175] Koelsch S. (2013).

[bb0180] Krumhansl C.L. (1998). Perceived triad distance: Evidence supporting the psychological reality of neo-Riemannian transformations. Journal of Music Theory.

[bb0185] Krumhansl C.L., Kessler E.J. (1982). Tracing the dynamic changes in perceived tonal organization in a spatial representation of musical keys. Psychological Review.

[bb0190] Kuhl P.K. (2004). Early language acquisition: Cracking the speech code. Nature Reviews Neuroscience.

[bb0195] Ladefoged P., Maddieson I. (1990). Vowels of the world’s languages. Journal of Phonetics.

[bb0200] Lagoudakis M.G., Parr R. (2003). Least-squares policy iteration. Journal of Machine Learning Research.

[bb0205] Lake B.M., Ullman T.D., Tenenbaum J.B., Gershman S.J. (2017). Building machines that learn and think like people. Behavioral and Brain Sciences.

[bb0210] Liebenthal E., Silbersweig D.A., Stern E. (2016). The language, tone and prosody of emotions: neural substrates and dynamics of spoken-word emotion perception. Frontiers in Neuroscience.

[bb0215] Lieck R. (2018).

[bb0220] Lieck R., Moss F.C., Rohrmeier M. (2020). The tonal diffusion model. Transactions of the International Society for Music Information Retrieval.

[bb0225] Little H., Eryılmaz K., de Boer B. (2017). Signal dimensionality and the emergence of combinatorial structure. Cognition.

[bb0230] MacKay D.J. (2003).

[bb0235] Marino L., Connor R.C., Fordyce R.E., Herman L.M., Hof P.R., Lefebvre L., Whitehead H. (2007). Cetaceans have complex brains for complex cognition. PLoS Biology.

[bb0240] Meyer L.B. (1956).

[bb0245] Milne A.J., Holland S. (2016). Empirically testing Tonnetz, voice-leading, and spectral models of perceived triadic distance. Journal of Mathematics and Music.

[bb0250] Moulin-Frier C., Nguyen S.M., Oudeyer P.Y. (2014). Self-organization of early vocal development in infants and machines: The role of intrinsic motivation. Frontiers in Psychology.

[bb0255] Moulin-Frier C., Oudeyer P. (2012). 2012 IEEE international conference on development and learning and epigenetic robotics (ICDL).

[bb0260] Murakami M., Kröger B., Birkholz P., Triesch J. (2015). 2015 joint IEEE international conference on development and learning and epigenetic robotics (ICDL-EpiRob).

[bb0265] Nolfi S., Mirolli M. (2010). Evolution of communication and language in embodied agents.

[bb0270] Nöth W. (1990).

[bb0275] Nowak M.A., Krakauer D.C. (1999). The evolution of language. Proceedings of the National Academy of Sciences.

[bb0280] Ogden C.K., Richards I.A. (1923).

[bb0285] Oliphant M., Batali J. (1997). Learning and the emergence of coordinated communication. Center for Research on Language Newsletter.

[bb0290] Ouattara K., Lemasson A., Zuberbühler K. (2009). Campbell’s monkeys concatenate vocalizations into context-specific call sequences. Proceedings of the National Academy of Sciences.

[bb0295] Oudeyer P.Y. (2003). The production and recognition of emotions in speech: Features and algorithms. International Journal of Human-Computer Studies.

[bb0300] Oudeyer P.Y. (2005). The self-organization of speech sounds. Journal of Theoretical Biology.

[bb0305] Oudeyer P.Y., Kaplan F. (2007). Language evolution as a Darwinian process: Computational studies. Cognitive Processing.

[bb0310] Patel A.D. (2003). Language, music, syntax and the brain. Nature Neuroscience.

[bb0315] Patel A.D. (2010).

[bb0320] Pearce M., Rohrmeier M. (2012). Music cognition and the cognitive sciences. Topics in Cognitive Science.

[bb0325] Peirce C.S. (1974).

[bb0330] Piantadosi S.T., Tily H., Gibson E. (2011). Word lengths are optimized for efficient communication. Proceedings of the National Academy of Sciences.

[bb0335] Rendell L., Whitehead H. (2001). Culture in whales and dolphins. Behavioral and Brain Sciences.

[bb0340] Riemann H. (1896).

[bb0345] Roberson D., Davidoff J., Davies I.R.L., Shapiro L.R. (2004). The development of color categories in two languages: A longitudinal study. Journal of Experimental Psychology: General.

[bb0350] Robert C.P., Casella G. (2004).

[bb0355] Robins R.H. (2014).

[bb0360] Sandhofer C.M., Smith L.B. (1999). Learning color words involves learning a system of mappings. Developmental Psychology.

[bb0365] Scherer K.R. (2003). Vocal communication of emotion: A review of research paradigms. Speech Communication.

[bb0370] Schröder M. (2001). Seventh European conference on speech communication and technology.

[bb0375] Sethares W.A. (2005).

[bb0380] Shannon C.E. (1948). A mathematical theory of communication. Bell System Technical Journal.

[bb0385] Shannon C.E. (1959). Coding theorems for a discrete source with a fidelity criterion. IRE Nat. Conv. Rec.

[bb0390] Shepard R.N. (1964). Circularity in judgments of relative pitch. The Journal of the Acoustical Society of America.

[bb0395] Spranger M. (2016).

[bb0400] Steels L. (1997). The synthetic modeling of language origins. Evolution of Communication.

[bb0405] Steels L. (1998). Synthesising the origins of language and meaning using co-evolution, self-organisation and level formation. Approaches to the Evolution of Language.

[bb0410] Steels L., Belpaeme T. (2005). Coordinating perceptually grounded categories through language: A case study for colour. Behavioral and Brain Sciences.

[bb0415] Sutton R.S., Barto A.G. (2018).

[bb0420] Tenenbaum J.B., De Silva V., Langford J.C. (2000). A global geometric framework for nonlinear dimensionality reduction. Science.

[bb0425] Vallabha G.K., McClelland J.L., Pons F., Werker J.F., Amano S. (2007). Unsupervised learning of vowel categories from infant-directed speech. Proceedings of the National Academy of Sciences.

[bb0430] Van Eecke P., Beuls K. (2020). AAAI spring symposium: Challenges and opportunities for multi-agent reinforcement learning.

[bb0435] Wilbrecht L., Nottebohm F. (2003). Vocal learning in birds and humans. Mental Retardation and Developmental Disabilities Research Reviews.

[bb0440] Wittgenstein L. (1953).

[bb0445] Yurk H., Barrett-Lennard L., Ford J.K.B., Matkin C.O. (2002). Cultural transmission within maternal lineages: Vocal clans in resident killer whales in southern Alaska. Animal Behaviour.

[bb0450] Zaslavsky N., Kemp C., Regier T., Tishby N. (2018). Efficient compression in color naming and its evolution. Proceedings of the National Academy of Sciences.

[bb0455] Zuidema W., Westermann G. (2003). Evolution of an optimal lexicon under constraints from embodiment. Artificial Life.

