# Supplementary material for: Discretisation and continuity: The emergence of symbols in communication
Source: Cognition. 2021 Oct;215:104787. doi: 10.1016/j.cognition.2021.104787 (PMC8381766; doi:10.1016/j.cognition.2021.104787)
Supplement: Supplementary file 1 — Supplementary material [file mmc1.docx]

<https://robert-lieck.github.io/emergence-of-symbols>, archived at Zenodo with DOI [10.5281/zenodo.4717800](http://dx.doi.org/10.5281/zenodo.4717800)
